# Modulating Iron Crystals with Lattice Chalcophile‐Siderophile Elements for Selective Dechlorinations Over Hydrogen Evolution

**DOI:** 10.1002/advs.202416663

**Published:** 2025-03-07

**Authors:** Xiaohong Hu, Qianhai Zhou, Du Chen, Zhongyuan Guo, Yiman Gao, Chaohuang Chen, Jie Hou, Vincent Noël, Daohui Lin, Lizhong Zhu, Jiang Xu

**Affiliations:** ^1^ College of Environmental and Resource Sciences Zhejiang University Hangzhou 310058 China; ^2^ Zhejiang Provincial Key Laboratory of Organic Pollution Process and Control Zhejiang University Hangzhou 310058 China; ^3^ Stanford Synchrotron Radiation Lightsource SLAC National Accelerator Laboratory Menlo Park CA 94025 USA

**Keywords:** chalcophile‐siderophile elements, iron microenvironment, lattice engineering, selective dechlorination

## Abstract

Selective dechlorination of organic chlorides over hydrogen evolution reaction (HER) remains a challenge because of their coincidence. Nanoscale zerovalent iron (nFe^0^) draws a promising picture of in situ groundwater dechlorination, but its indiscriminate reactivity limits the application. Here, nFe^0^ crystals are designed with electron shuttles and improved hydrophobic nature based on elemental chalcophile‐siderophile characteristics, where chalcophile‐siderophile S served as a bridge to allow impregnating nFe^0^ crystals with weakly siderophile and strongly chalcophile Cu. Even impregnations of lattice chalcophile‐siderophile elements into the nFe^0^ crystals are evidenced at both intraparticle and individual‐particle levels. The modulated Fe microenvironment and physicochemical properties broke the reactivity‐selectivity‐longevity‐stability trade‐off. Compared to nFe^0^, superhydrophobic Cu─S─nFe^0^ with lattice expansion promoted dechlorination by 20‐fold but inhibited HER by 150‐fold, utilizing ≈80–100% electrons from the Fe^0^ reservoir. This work demonstrates the concept of engineering nFe^0^ lattice with tunable structure‐property relationships, mimicking reductive dehalogenases by selectively interacting with halocarbon functional groups for efficient dehalogenation and sustainable groundwater remediation.

## Graphical abstract

Lattice engineering of nFe^0^ crystals with chalcophile‐siderophile elements optimizes the iron microenvironment, enhancing hydrophobicity, electron transfer, and selective dechlorination efficiency while reducing hydrogen evolution reaction (HER). The Cu─S─nFe^0^ material shows a 20‐fold increase in dechlorination rate, a 150‐fold decrease in HER, and nearly 100% electron utilization, offering a cost‐effective solution for groundwater remediation.

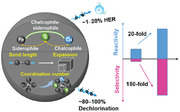

## 1 Introduction

Organic chlorides have been broadly used as bulk commodity chemicals in industry and agriculture, e.g., chlorinated solvents and chlorinated antibiotics.^[^ ^1^ ^]^ Their production and utilization at the thousands of tons level inevitably release them into the groundwater,^[^ ^1^, ^2^ ^]^ which is a vital resource of freshwater for drinking water supply and agricultural irrigation. Numerous efforts have been devoted to addressing this global health and environmental concern by developing technologies for dechlorination.^[^ ^3^ ^]^ However, the dechlorination of organic chlorides is usually energy‐intensive or needs long reaction times, often leading to non‐selective reactions and being limited by competing reactions, especially the hydrogen evolution reaction (HER).^[^ ^4^ ^]^ An ideal design material for economical and sustainable groundwater remediation should make the dechlorination outcompete HER.

Water is the largest electron acceptor during groundwater remediation or water treatment, which aims to treat the target contaminant in water but not the water itself. Excessive capacity of materials would be consumed if the competing HER is not blocked. Nanoscale zerovalent iron (nFe^0^) with environmental friendliness has been proposed as a promising technology for in situ groundwater dechlorination for ≈3 decades.^[^ ^5^ ^]^ However, academic and industrial interests in this material are being lost, because of its low selectivity for dechlorination (usually <2% electron utilization), short longevity in water (typically <60 days), and poor stability during transport and storage.^[^ ^6^ ^]^ Ultimately, nFe^0^ particles readily react with water, and the surface passivation by the generated iron (hydr)oxides from the HER makes the situation even worse.^[^ ^7^ ^]^ Overwhelming protons tend to accumulate on the surface of transition‐metal‐based materials like nFe^0^, which usually favor the competing HER over the dechlorination of organic chlorides.^[^ ^8^ ^]^ In light of this aspect, it is highly desired to regulate the mass transfer and diffusion processes of electrons, protons, and organic chlorides at the interface of nFe^0^ materials to achieve efficient and selective dechlorinations.

Engineering the crystal structure and coordination environment of iron‐based materials is an emerging strategy to regulate their proton and electron transfer, breaking the reactivity selectivity trade‐off.^[^ ^9^ ^]^ The reactivity and selectivity of Fe sites can be promoted by modulating their interactions with nonmetal *p*‐block elements and transition‐metal *d*‐block elements, e.g., iron sulfides and bimetals with surface‐doped Cu.^[^ ^10^ ^]^ Both *d*‐*d* and *p*‐*d* orbital hybridizations are expected to tune the *d*‐band center of Fe and its Fermi level, inducing electron redistribution, changing their interactions with ^*^H, and affecting electron transfer.^[^ ^11^ ^]^ Selective reductions of CO_2_, N_2_, and NO_3_^−^ have been well established for iron minerals, bimetals, and single‐atom catalysts.^[^ ^12^ ^]^ Previously reported Cu addition to nFe^0^ or S─Fe^0^ materials have improved the reactivity of materials, despite Cu was doped on the surface. However, impregnating nFe^0^ crystals via lattice engineering for selective dechlorination over HER mainly stays at the conceptual stage. One important question is how to rationally and evenly distribute *p*‐block and *d*‐block elements into the crystals of nFe^0^. Ni and S have been recently incorporated into nFe^0^ crystals via Lewis acid‐base interactions for selective reductions of organic contaminants over water.^[^ ^13^ ^]^ While Ni is best known as a hydrogenation catalyst, it is unclear if incorporating an electron shuttle (i.e., Cu) will change the story. In addition, Cu is relatively cheaper and less toxic, more S is expected to be incorporated into the materials due to the chalcophile‐siderophile interactions, and the lower band gap and higher hydrophobicity of copper sulfides (e.g., CuS_2_ and CuFeS_2_) are also beneficial for selective dechlorination over HER.^[^ ^14^ ^]^

This work describes the discovery of the first superhydrophobic lattice‐engineered Cu─S─nFe^0^ material to address the abovementioned challenges. A feasible strategy was developed to evenly distribute lattice Cu and S into nFe^0^ crystals based on their chalcophile‐siderophile characteristics,^[^ ^15^ ^]^ unlike traditional doping methods, where the elements typically exist on the surface or are unevenly distributed over the particles. Based on the chalcophile‐siderophile theory, while strongly siderophile Co could be feasibly incorporated into the nFe^0^ crystals, the weakly siderophile Cu could not.^[^ ^16^ ^]^ Chalcophile‐siderophile S served as a bridge to allow the impregnation of nFe^0^ crystals with weakly siderophile and strongly chalcophile Cu.^[^ ^16^, ^17^ ^]^ The S/Fe molar ratio was quantified using ICP‐OES (Agilent 5110) after aqua regia digestion, similar to the Cu(Co)/Fe molar ratio. Details can be found in the Supporting Information. These chalcophile‐siderophile elements with a unique structure successfully tuned the local microenvironment of Fe atoms and optimized the physicochemical properties of materials, which were extensively characterized. The lattice expansion, coordination environment, speciation, electron transfer ability, and hydrophobicity explained well the satisfactory water longevity, air stability, and efficient and selective dechlorination over HER. This developed lattice engineering strategy is operationally simple with a limited increase in material production cost compared to nFe^0^, providing immense potential for breaking reactivity‐selectivity‐longevity‐stability trade‐offs.

## 2 Results and Discussion

### 2.1 Single‐Particle Distributions of nFe^0^ with Lattice‐Doped Chalcophile‐Siderophile Elements

**Figure 1:**
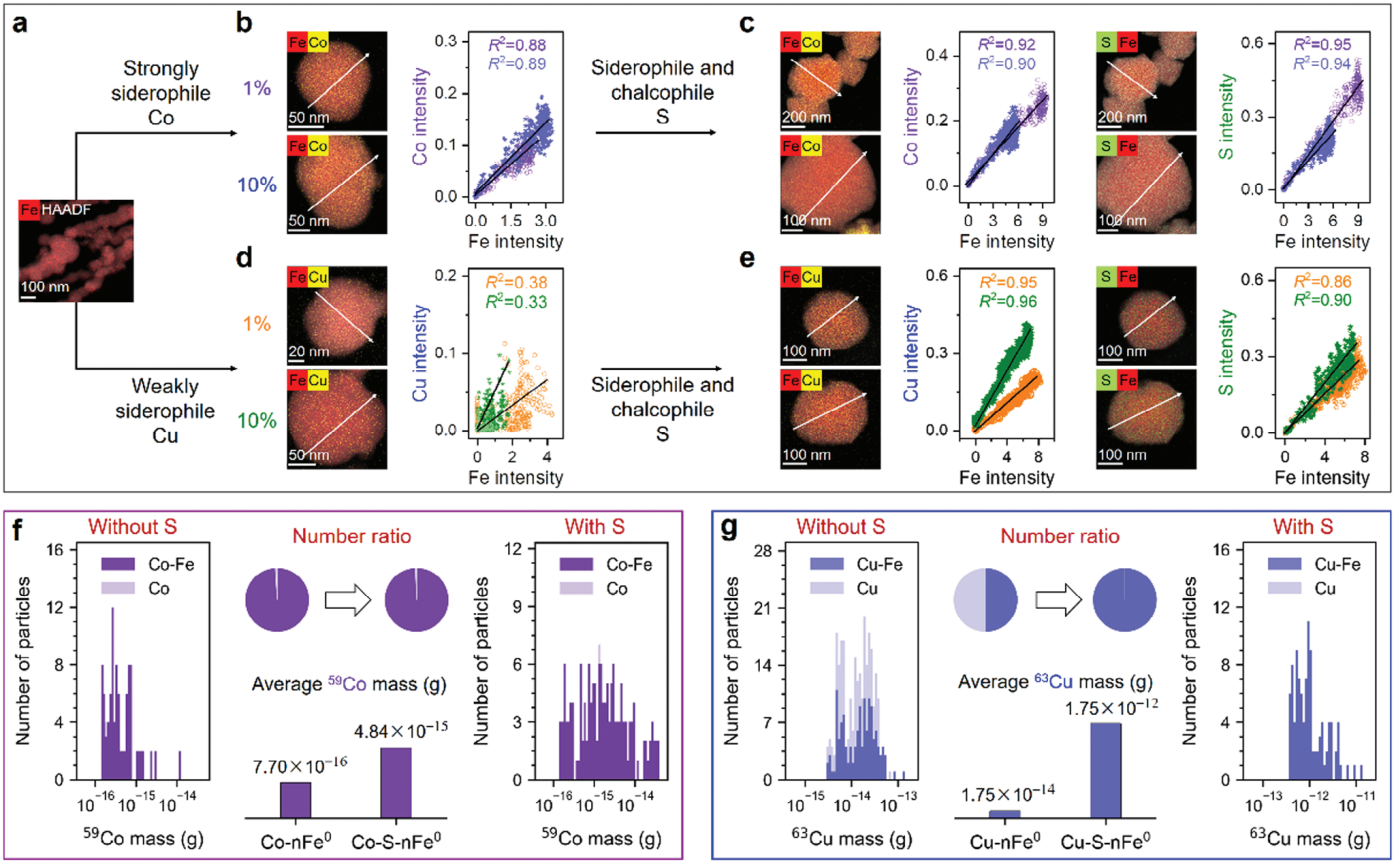
Single‐particle distributions of nFe^0^ with lattice‐doped chalcophile‐siderophile elements. Morphology and intraparticle elemental distribution of a) pristine nFe^0^, b,c) Co‐doped nFe^0^ and d,e) Cu‐doped nFe^0^ in the absence or presence of lattice S ([Co/Fe]_mol_ and [Cu/Fe]_mol_ = 1% and 10%). Individual‐particle‐level associations of Fe with f) Co and g) Cu in the absence or presence of lattice S ([Co/Fe]_mol_ and [Cu/Fe]_mol_ = 1%).

The distributions of Fe and incorporated chalcophile‐siderophile elements at the intraparticle level were measured to understand their interactions in the materials. High‐angle annular dark‐field (HAADF) images with high probabilities of elemental co‐associations in the line scans (*R* ^2^ = 0.88–0.95) and the absence of Co peaks in the X‐ray diffraction (XRD) patterns (**Figure** **1**a–c; Figure S1, Supporting Information) suggest that the strongly siderophile Co was evenly distributed with Fe, regardless of the chalcophile S incorporation or Co content (1% or 10%). In contrast, the probabilities of Fe─Cu co‐association in the line profile of Cu─nFe^0^ were relatively low, i.e., *R* ^2^<0.40 (Figure 1d), likely due to the low siderophile affinity of Cu.^[^ ^16a,c^ ^]^ However, the incorporation of chalcophile S significantly enhanced the intensity of Cu and its co‐association probabilities with Fe, and all the elements were distributed homogeneously throughout the particles (*R* ^2^ = 0.87–0.99) rather than concentrated on the surface (Figure 1e). This was probably because of the role of S in the budget and partitioning of chalcophile and siderophile elements,^[^ ^16^, ^18^ ^]^ where the affinities of S with Fe and Cu were both high and S could be favorably incorporated into nFe^0^ particles (Figure S2, Supporting Information).^[^ ^13^, ^19^ ^]^ These uniform distributions indicate that the doped Co, Cu, or S might have entered the crystals of nFe^0^ particles.

The multi‐elemental composition was further characterized by the single‐particle inductively coupled plasma time‐of‐flight mass spectrometry (spICP‐TOF‐MS) to assess the impact of S on the co‐association of metals at the individual‐particle level. Like the above intraparticle distribution, ≈100% the Co‐bearing particles were associated with Fe for Co─nFe^0^ and Co─S─nFe^0^ materials (Figure 1f). About half of the Cu‐bearing particles were recognized as “Cu only” particles for Cu─nFe^0^ material (Figure 1g), i.e., either associated with limited Fe mass (below the detection limit, i.e., ≈10^−4^ ppm (LOD IUPAC)) or present as Cu^0^ nanoparticles. Interestingly, 100% of the Cu‐bearing particles were associated with Fe after incorporating chalcophile S, and the average Cu mass of Cu─S─nFe^0^ particles was significantly higher than that of Cu─nFe^0^ particles. This increased Cu density of individual particles after S doping was consistent with the increased particle size, decreased surface area, and slightly decreased Fe^0^ content (Figure S3, Supporting Information). While the regular ICP‐MS after acid digestion only verified that all the added Co and Cu could be utilized during materials synthesis (Figure S4, Supporting Information), these distributions at single‐particle levels provide unprecedented resolution of how chalcophile‐siderophile elements associated with nFe^0^ particles and changed their microenvironments. The incorporation of Co, used primarily as a siderophile reference for comparison, underscores the contrasting behavior of Cu and S. While Co readily incorporated into the Fe lattice due to its strong siderophile affinity, this study focuses on the challenges associated with Cu incorporation and the critical role of S in overcoming these limitations. Consequently, the structural and functional effects of Co are not explored further in this work. The most distinguished point or novel concept of chalcophile‐siderophile modulation is that this strategy can evenly incorporate chalcophile‐siderophile elements into crystals. Generally, Cu is challenging to integrate into the Fe lattice as shown in Figure 1, but can be uniformly distributed due to the synergistic effect of S. The proposed concept of “chalcophile‐siderophile modulation” builds on this unique behavior, exploring how co‐doping elements like Cu and S modulate the structure and performance of Fe^0^‐based materials. Unlike traditional sulfidation strategies that mainly affect surface interactions, this approach facilitates crystal‐level modifications, enhancing not only the hydrophobicity but also the material's electronic and properties, as discussed below.

### 2.2 Tunable Fe Microenvironment by Lattice Chalcophile‐Siderophile Elements

**Figure 2:**
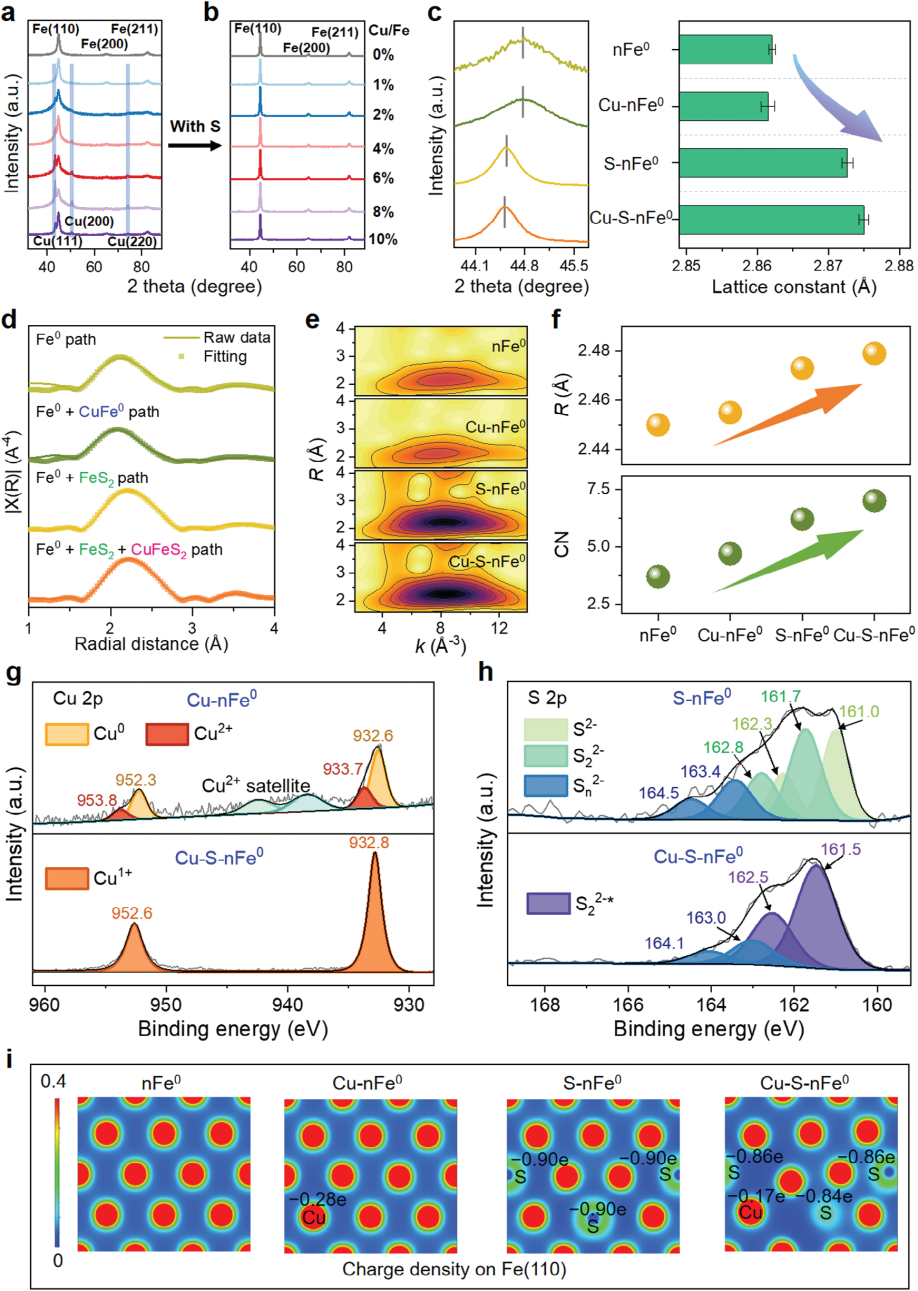
Tunable Fe microenvironment by lattice chalcophile‐siderophile elements. XRD patterns of Cu‐doped nFe^0^ a) with S or b) without S. c) Shifts of Fe(110) diffraction peak and relevant lattice constants. d) Fe K‐edge Fourier‐transformed EXAFS spectra and their best shell fits, e) wavelet transform, and f) radial distance (*R*) and coordination number (CN) derived from the shell fits at the @Fe.1@ site on the first shell. g) Cu 2p and h) S 2p XPS spectra of lattice‐doped nFe^0^ materials. i) DFT‐calculated charge density on the Fe(110) surface of lattice‐doped nFe^0^ materials.

The distributions of Fe and incorporated chalcophile‐siderophile elements at the intraparticle level were The crystal structures were evaluated by XRD and the emerged Cu^0^ peaks suggest that Cu could not be well incorporated into the Fe crystals (**Figure** **2**a), countering the strongly siderophile Co where no extra peak was observed except for Fe peaks (Figure S1, Supporting Information). The relatively clear Fe peaks with good crystallinity of Cu─S─nFe^0^ particles (Figure 2b) indicate that both Cu and S could be incorporated into nFe^0^ body‐centered‐cubic (bcc) structure due to the interactions between these chalcophile‐siderophile elements.^[^ ^15^, ^16^, ^17^ ^]^ This was in line with the fact that Cu and S were evenly distributed throughout the nanoparticles (Figure 1e) and the increase of lattice S amount in the presence of lattice Cu (Figure S5, Supporting Information). Moreover, the incorporation of a small amount of lattice Cu ([Cu/Fe]_mol_ = 1%) and S ([S/Fe]_mol_ = 5–8%) provoked the shift of Fe peaks to a lower angle, indicating a phase‐segregated or alloy structure as well as the lattice expansion of Fe crystals (Figure 2c).^[^ ^13^, ^20^ ^]^ Although the Cu‐induced lattice expansion of nFe^0^ crystal was smaller than that of S, the further increased lattice constant of Cu─S─nFe^0^ indicated its faster electron transfer since the interatomic distance is inversely proportional to the bandgap.^[^ ^21^ ^]^

The Fe coordination environments in the materials were identified at an atomic level by the Fe K‐edge extended X‐ray absorption fine structure (EXAFS) analyses. The shell fits of Cu─nFe^0^ and S─nFe^0^ were slightly and significantly improved by adding the scatting paths of Fe─Cu and Fe─S, respectively (Figure 2d; Figure S6, Supporting Information), providing another proof of poor Cu incorporation and good lattice S incorporation. The Fe─S paths of pyrite (FeS_2_) fitted better than those of mackinawite (FeS), suggesting that the lattice S was present as a FeS_2_‐like structure in the S─nFe^0^ materials, similar to recently reported lattice S doped versions.^[^ ^19b,c^ ^]^ More intriguingly, the addition of Fe─Cu and Cu─S scatting paths from chalcopyrite (CuFeS_2_) presented a better shell fit for Cu─S─nFe^0^, suggesting a CuFeS_2_‐like structure along with the Fe bcc structure. These results also indicate that incorporating lattice S and Cu as different crystal unit cells of FeS_2_ (fcc, *a* = 5.40 Å) and CuFeS_2_ (fcc, *a* = 5.23 Å) into Fe^0^ (bcc, *a* = 2.85 Å) could induce lattice expansions.^[^ ^19^, ^22^ ^]^ Combined with the above elemental maps at single‐particle levels (Figure 1) and crystal structures of nFe^0^ (Figure 2a–c), these shell fits with varied crystallinity further evidenced the interactions of Fe with lattice chalcophile‐siderophile elements. The wavelet transform contour plots in both K and R space of Fe K‐edge EXAFS also revealed the impacted coordination environments (Figure 2e). More atoms surrounded the central Fe after incorporating lattice Cu and S (Figure 2f; Table S1, Supporting Information), according to the increased coordination number (CN), i.e., from 3.7 to 7.0 in the first shell. Meanwhile, the increase in bonding length (*R*) was also consistent with the increased lattice constant (Figure 2c).

The surface species of lattice‐doped nFe^0^ materials were measured by X‐ray photoelectron spectroscopy (XPS). It is noteworthy that the Cu 2p peak and S 2p peak shifted to higher binding energies after incorporating lattice S and Cu, respectively (Figure 2g,h), indicating the partial electron transfer from the Cu or S to Fe.^[^ ^23^ ^]^ According to the absence of Cu^2+^ satellites in the Cu─S─nFe^0^, the main valence state of Cu was probably monovalent rather than divalent or zero‐valent.^[^ ^24^ ^]^ Likewise, S_2_^2‐*^ species of CuFeS_2_ structure were detected based on the pattern characteristics and the position of S peaks,^[^ ^25^ ^]^ coinciding with the shell fitting results of Cu─S─nFe^0^ (Figure 2d). The charge density distribution on Fe(110) in the presence of lattice Cu and S (Figure 2i) further confirmed the change of electron density, which could lead to the shift of binding energies (Figure 2g,h). Although the effect of lattice Cu on charge redistribution is less than that of lattice S on the Fe(110) surface, both lattice Cu and S attract electrons from nearby Fe sites, as indicated by the relatively negative Bader charge (Figure 2i). Consequently, the Cu and S sites can become less attractive for *H adsorption and contribute to (super)hydrophobicity of (Cu‐)S─nFe^0^, as discussed below.

It has to be acknowledged that XPS provides a macroscopic, surface‐sensitive snapshot of the material's electronic environment, while DFT calculations offer a more detailed, atomistic‐level description of the electronic interactions. Both results are complementary and indicate that Cu and S doping indeed modifies the electronic properties of Fe^0^, but they reflect different perspectives on the underlying electronic changes. This difference does not indicate a contradiction but rather highlights the distinct perspectives of experimental and theoretical analyses. While charge redistribution between two metals has been well established,^[^ ^26^ ^]^ these results suggest that lattice doping chalcophile‐siderophile elements into nFe^0^ crystals could also alter their electronic structure, assuming to possess a better electron transfer ability. In addition, combined with the facts of low surface Fe^0^ contents on S─nFe^0^ and Cu─S─nFe^0^ materials (Figure S7, Supporting Information) and the absence of surface oxidized S species (e.g., SO_4_^2−^) (Figure 2h), these materials were well covered by hydrophobic FeS_2_‐like and CuFeS_2_‐like species accompanied by inevitably present iron (hydr)oxides (Figure S8, Supporting Information). Hence, both the core and shell of nFe^0^ had been engineered. These altered microenvironments of nFe^0^ materials by lattice doping of chalcophile‐siderophile elements provided a feasible means to optimize their unique physicochemical properties, as described below.

### 2.3 Optimized Physicochemical Properties by Lattice Chalcophile‐Siderophile Elements

**Figure 3:**
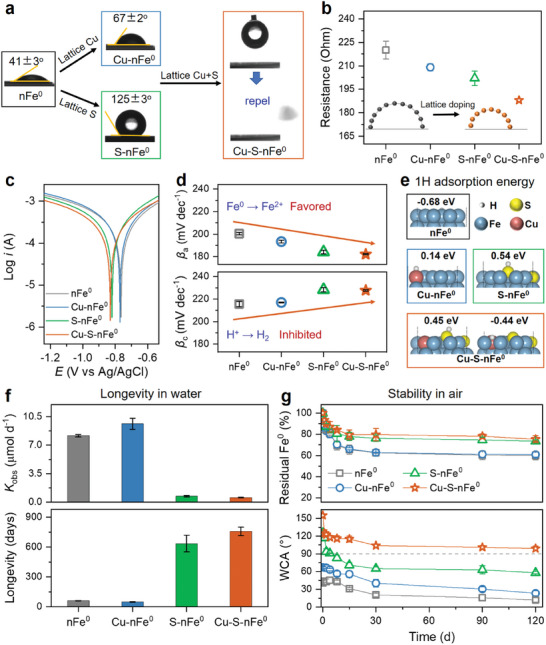
Optimized physicochemical properties by lattice chalcophile‐siderophile elements. a) Water contact angle (WCA) on dry pellets of lattice‐doped nFe^0^ materials. Electrochemical analysis of pellets made from lattice‐doped nFe^0^ materials, including b) fitted resistance, c) Tafel plots, and d) cathodic and anodic slopes. e) DFT‐calculated H adsorption energy on the Fe(110) surface. f) Longevity of materials in water assessed by hydrogen evolution rate and reactive lifetime. g) Stability of materials in air assessed by residual Fe^0^ content and WCA measurements. Data are presented as mean ± s.d. (*n* = 3).

The differences in elemental speciation and crystal structure affected the hydrophobicity and electron transfer of lattice‐doped nFe^0^. The measured water contact angles were 41±3° and 67±2° for the pellets made from nFe^0^ and Cu─nFe^0^ materials, respectively (**Figure** **3**a). The incorporation of FeS_2_‐like species endowed the S─nFe^0^ with a high hydrophobicity (>120°).^[^ ^6a^ ^]^ Most strikingly, the co‐incorporation of lattice Cu and S into nFe^0^ crystals induced a superhydrophobicity, presenting a strong water droplet repulsion (Video S1, Supporting Information). This phenomenon is consistent with the presence of CuFeS_2_‐like species, which is more hydrophobic than FeS_2_‐like species.^[^ ^14c^ ^]^ The electron‐transfer resistance of materials was probed by electrochemical impedance spectroscopy (EIS). Thanks to the electron shuttle effect of Cu and strong electronegativity nature of S,^[^ ^27^ ^]^ both the incorporations of lattice Cu and S significantly decreased the fitted resistance (Figure 3b; Figure S9, Supporting Information), confirming the improved electron transfer after lattice doping. This was consistent with the enlarged lattice constant and changed elemental speciation (Figure 2), where the band gap of CuFeS_2_ (0.35 eV) was lower than those of FeS_2_ (0.95 eV) and iron oxides (e.g., 2.20 eV for Fe_2_O_3_).^[^ ^14b,c^ ^]^ The complete transformation in hydrophobicity and reduced resistance indicates that the role of iron oxides is minimal compared to sulfur species after lattice doping with Cu and S. Tafel analysis was further conducted to determine if iron corrosion or hydrogen evolution (HER) was the favorite process (Figure 3c). The measured values of the anode slope (<200 mV dec^−1^) were relatively lower than those of the cathode slope (>210 mV dec^−1^) for each material (Figure 3d), indicating the tendency of iron corrosion to release electrons rather than hydrogen evolution.^[^ ^28^ ^]^ The increased adsorption energy of ^*^H on the lattice Cu and S sites correlated with the charge distribution (Figures 3e and 2i), indicating a weakened interaction between ^*^H and the surface of Fe(110). That is also responsible for both the suppression of hydrogen evolution and the (super)hydrophobic properties of (Cu‐)S─nFe^0^ (Figure 3a,d). Compared to the electronic structure modulation, the role of superhydrophobicity was expected to be more significant to the enhancement of material performance, especially selectivity for dechlorination over HER. These unique physicochemical properties would make the highly selective anaerobic dechlorinations over HER possible, as discussed later.

The benefit of lattice doping in the longevity of nFe^0^ materials was estimated by monitoring H_2_ evolution (Figure 3f; Figure S10, Supporting Information). H_2_ was quickly generated via the nFe^0^ corrosion with water, and lattice Cu promoted this reaction by ≈1.2 times. In contrast, lattice S and its co‐doping with Cu greatly inhibited the H_2_ evolution by 11.3–14.4 times, because of the FeS_2_‐like and CuFeS_2_‐like structure induced (super)hydrophobicity (Figure 3a) and S‐blocked H adsorption on nearby Fe sites.^[^ ^29^ ^]^ Therefore, the estimated longevity of Cu─S─nFe^0^ in water was much longer than other materials. Note that the H_2_ evolution could be slowed as the accumulation of iron (hydr)oxides on the surface, the estimated longevity should be at least 2 years. Similarly, the improved air stability of nFe^0^ after co‐doping lattice chalcophile‐siderophile elements was evidenced by the monitored hydrophobicity and residual Fe^0^ content (Figure 3g). While other materials lost hydrophobicity or became more hydrophilic, the Cu─S─nFe^0^ material maintained hydrophobic (≈100°) after exposure to air for 120 days, and the remained Fe^0^ content was ≈80% of the original value. Generally, the storage conditions of nFe^0^ materials are strict due to poor water and air stability, e.g., being stored in a vacuum package (dry particles that easy to “burn” once exposed to air) or ethanol (a suspension that needs pretreatments), or stabilized with an inert shell that dramatically decreasing their reactivity and selectivity toward target contaminants. However, these enhanced water longevity and air stability could greatly simplify material storage and transportation with reduced relevant costs, breaking the reactivity‐selectivity‐longevity trade‐off.

### 2.4 Broken Reactivity‐Selectivity Trade‐Off of nFe^0^ by Lattice Engineering

**Figure 4:**
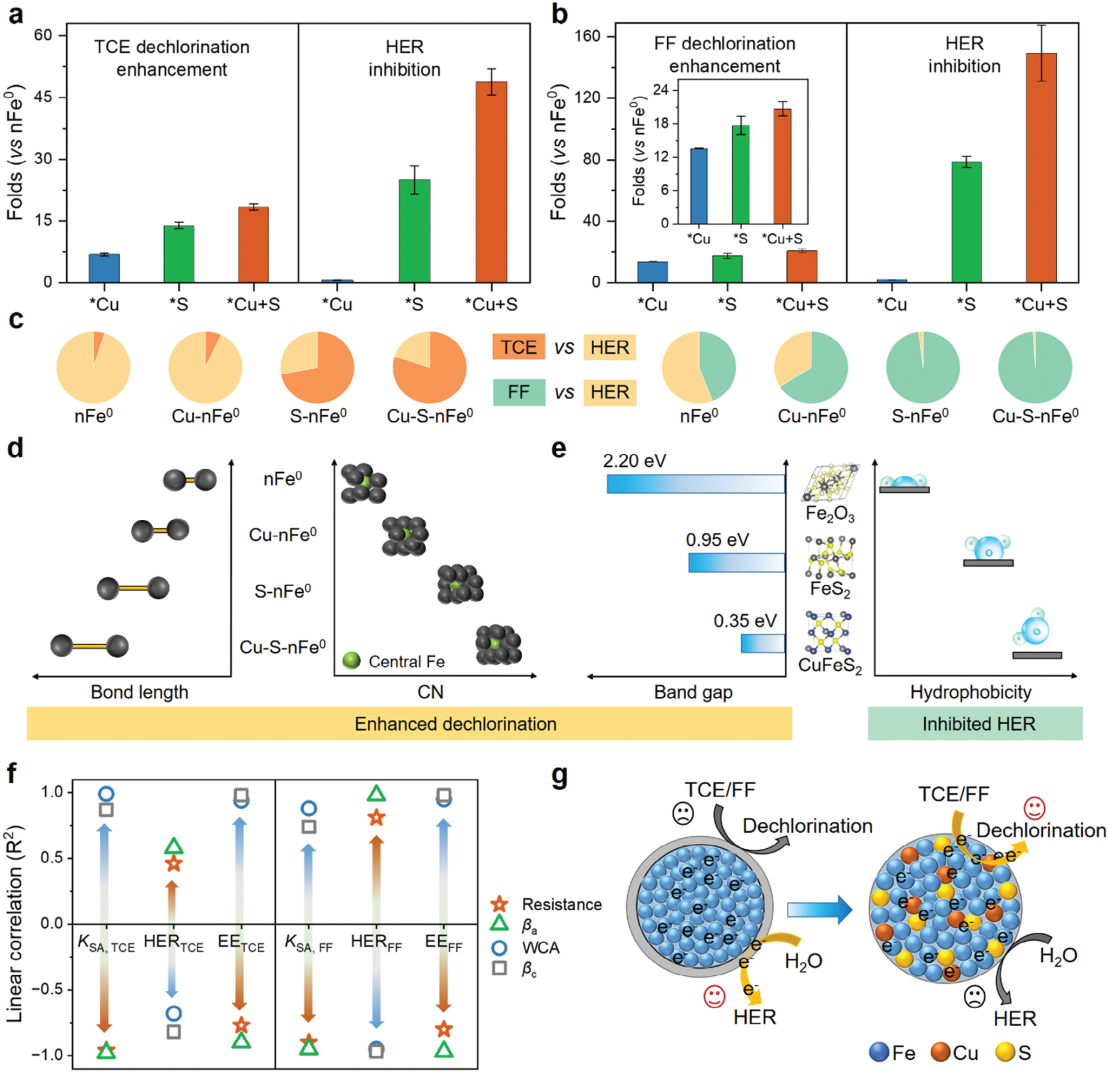
Broken reactivity‐selectivity trade‐off of nFe^0^ by lattice engineering. Dechlorination enhancements and HER inhibitions during the a) TCE and b) FF removals by of lattice‐doped materials relative compared to the unmodified nFe^0^ version. c) Electron selectivity of TCE and FF dechlorination over HER by lattice‐doped materials. (Basic conditions: 1 g L^−1^ materials, 70 µm TCE or 0.15 mM FF, pH = 6.5, 25 °C). Data are presented as mean ± s.d. (*n* = 3). d,e) Analysis of structure‐properties‐reactivity‐selectivity correlations, including bond length, coordination number, bandgap, and hydrophobicity. f) Linear correlations between properties indicators (resistance, *β*_a_, WCA, and *β*_c_) and dechlorination reactivity (*K*_SA_), H_2_ evolution (HER), and electron efficiency (EE). g) Illustration of selective dechlorination of TCE/FF by lattice Cu and S doped nFe^0^ particles in water.

Good water longevity and air stability are beneficial but only if the materials are still reactive with the target groundwater contaminants. Typical chlorinated solvent (trichloroethylene, denoted as TCE) and antibiotics (florfenicol, denoted as FF) were selected as reactivity‐selectivity probes.^[^ ^1b,c^ ^]^ After 120 days of stability testing (e.g., being exposed in air), the Cu─S─nFe^0^ material maintained removal efficiencies comparable to its fresh version, exhibiting relatively more stable and effective performance for TCE and FF dechlorination than other materials (Figure S11, Supporting Information). The *K*_SA,TCE_ of TCE dechlorination of Cu─nFe^0^, S─nFe^0^, and Cu─S─nFe^0^ were 7, 14, and 18 times higher than that of nFe^0^, respectively (**Figure** **4**a; Figure S12, Supporting Information). Similarly, both lattice Cu and S promoted the dechlorination of FF (Figure 4b; Figure S13, Supporting Information), and no Cu or S was leached from the particles after 7 days and 30 days of reaction, with few leachable Fe (<1%) (Figure S14, Supporting Information). Although water is the largest electron acceptor during water treatment or groundwater remediation, co‐doping of lattice Cu and S into nFe^0^ crystals successfully inhibited the HER by 49–150 times during the TCE and FF dechlorinations. Note that the competing HER consumed huge fractions of electrons from the Fe^0^ reservoir for TCE (95%) and FF (45%) dechlorinations. These values were dramatically decreased to 20% and ≈1%, respectively, after impregnating nFe^0^ crystals with chalcophile‐siderophile Cu and S (Figure 4c). These superior dechlorination reactivities with quite inert HER reactivity and high electron utilization efficiency suggest the broken reactivity‐selectivity trade‐off of nFe^0^ by lattice engineering.

These attractive efficient and selective dechlorinations over H_2_ evolution by lattice‐doped nFe^0^ materials were corroborated well with the improved hydrophobicity and electron transfer (Figure 3), which allowed their inhibited water reactivity but facilitated contaminant reductions. These crucial physicochemical properties of lattice‐doped nFe^0^ materials could be modulated by changing the crystal structure and speciation (Figure 4d,e). Co‐doping Cu and S into the nFe^0^ lattice as relatively larger crystal systems (e.g., fcc FeS_2_ and tetragonal CuFeS_2_) could cause lattice strain, increasing the coordination numbers of central Fe atoms and their bond length. The empirical relation between energy gap and lattice constant in cubic semiconductors would help to estimate the impacted electron transfer ability of nFe^0^ by lattice engineering.^[^ ^21b^ ^]^ Moreover, the newly incorporated FeS_2_‐like and CuFeS_2_‐like structures possessed a lower band gap and higher hydrophobicity than iron oxides that typically formed on unmodified nFe^0^ materials. This would advance the design of lattice‐doped nFe^0^ materials to break their reactivity‐selectivity‐longevity‐stability trade‐off, achieving efficient and selective dechlorinations over HER, as shown above.

Further linear correlation assessment showed that the dechlorination rate (*K*_SA_) and electron efficiency (EE) were positively correlated with WCA and *β*_c_, but negatively correlated with resistance and *β*_a_, in both the TCE and FF systems (Figure 4f; Figures S15–S18, Supporting Information). This suggests that the hydrophobicity, inhibited HER, lower resistance, and favored Fe^0^ corrosion are important properties for selective dechlorination of TCE and FF over HER, which was achieved by incorporating Cu and S into nFe^0^ lattice (Figures 3 and 4g). This was because lattice Cu and S both contributed to expanding Fe^0^ lattice, modulating the charge redistribution, and generating priority species (i.e., FeS_2_‐like and CuFeS_2_‐like species) (Figure 2), allowing Cu─S─nFe^0^ to more readily release electrons and selectively reduce target contaminants in water. The completely opposite positive/negative correlation results of HER, compared with *K*_SA_ and EE (Figure 4f; Figures S15–S18, Supporting Information), were consistent with the above conclusion. These results indicate that co‐doping Cu and S into nFe^0^ particles can facilitate selective dechlorination of TCE and FF over HER in the water matrix, which would further contribute to groundwater remediation.

### 2.5 Assessments of Application Potential for Groundwater Remediation

**Figure 5:**
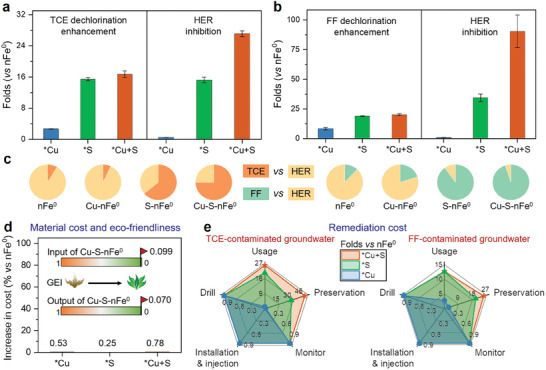
Assessments of application potential for groundwater remediation. Dechlorination enhancements and HER inhibitions during the a) TCE and b) FF removals from real groundwater by of lattice‐doped materials relative compared to the unmodified nFe^0^ version. c) Electron selectivity of TCE and FF dechlorination over HER by lattice‐doped materials in real groundwater. (Basic conditions: 1 g L^−1^ materials, 70 µm TCE or 0.15 mM FF, pH = 8.5, 25 °C). Assessments of d) material cost and eco‐friendliness and e) remediation cost for TCE and FF contaminated groundwater. Data are presented as mean ± s.d. (*n* = 3).

The application potential of nFe^0^ with lattice chalcophile‐siderophile elements was evaluated in real groundwater matrices. Enhanced TCE and FF dechlorinations (up to 17–21 folds), inhibited H_2_ evolution (up to 27–90 folds), and improved electron selectivity (up to 75–95%) of nFe^0^ materials were observed after lattice engineering, where the superhydrophobic Cu‐S‐nFe^0^ material with the fastest electron transfer provided the best performance (**Figure** **5**a–c; Figures S19 and S20, Supporting Information). Meanwhile, limited Cu and Fe (<1%) were leached from particles after ≈20 days of reaction (Figure S21, Supporting Information), and the leached amount of S (≈0.04 mg L^−1^) was negligible compared to the background of groundwater (≈9 mg L^−1^). This limited reactivity‐selectivity trade‐off in real groundwater matrix agreed well with those in deionized water.

The environmental friendliness during the synthesis process of Cu─S─nFe^0^ material was evidenced by the low input and output GEI values (0.099 and 0.070) from the Biwer‐Heinzle environmental evaluation (Figure 5d; Table S2, Supporting Information).^[^ ^30^ ^]^ Incorporating lattice Cu and S into the nFe^0^ crystals induced negligible increases in the material production cost (0.2–0.8%), as compared to unmodified nFe^0^. Moreover, these lattice chalcophile‐siderophile elements could greatly minimize the material usage (up to 26 and 13 folds) and preservation cost (up to 52 and 25 folds) due to the improved reactivity‐selectivity‐longevity‐stability of materials (Figure 5e; Table S3, Supporting Information). Thus, even assuming the drilling‐installation‐injection‐monitoring costs of using lattice‐doped nFe^0^ are similar to unmodified nFe^0^ (usually need multiple injections and a long monitoring period due to their poor performance), the remediation cost could be largely reduced. In summary, these assessments of application potential suggest that lattice engineering nFe^0^ materials with chalcophile‐siderophile elements could be a promising strategy for efficient and selective dechlorination over HER in groundwater.

## 3 Conclusion

This study demonstrates that engineering nFe^0^ crystals with lattice chalcophile‐siderophile elements is capable of solving the challenge of selective dechlorination over HER present in groundwater remediation. Extensive characterizations at intraparticle, individual particle, and atomic levels reveal that the chalcophile‐siderophile nature of Fe, Cu, and S allowed their homogenous distribution over the nFe^0^ particles, tuning the microenvironment of Fe sites and physicochemical properties of materials. In particular, the chalcophile nature of S facilitates the even incorporation of weak siderophile Cu into the Fe lattice. While S plays a dominant role in hydrogen evolution inhibition, the role of Cu cannot be overlooked as it significantly contributes to the overall performance of the material. The electron shuttle effects of Cu and S along with the improved hydrophobicity of their species endowed the materials with excellent performance for breaking reactivity‐selectivity‐longevity‐stability trade‐off. The superhydrophobic Cu─S─nFe^0^ material exhibited accelerated dechlorinations (up to 20‐fold) accompanied by inhibited HER (up to 150‐fold), providing 80% and nearly 100% electron utilization efficiency for TCE and FF dechlorinations, respectively. Optimizing the Cu and S ratios presents an intriguing avenue for further exploration, as it could unlock new strategies to refine material properties and enhance performance. Compared to nFe^0^, the simple synthesis process of Cu─S─nFe^0^ showed a negligible increase in material production cost but a remarkable decrease in groundwater remediation cost, suggesting its high application potential. While element‐doping is a broad concept referring to introducing any element into a material to modify its properties, chalcophile‐siderophile modulation represents a more specialized strategy that leverages the specific affinities between elements. These findings provide a foundation for lattice engineering nFe^0^ crystals with nonmetal *p*‐block elements and transition‐metal *d*‐block elements, constructing high‐performance materials for efficient and selective groundwater remediation.

## 4 Experimental Section

All experimental details can be found in the Supporting Information.

## Conflict of Interest

The authors declare no conflict of interest.

## Supporting information



Supporting Information

Supplemental Video 1

## Data Availability

The data that support the findings of this study are available from the corresponding author upon reasonable request.
