# Supplementary material for: Modulating Iron Crystals with Lattice Chalcophile‐Siderophile Elements for Selective Dechlorinations Over Hydrogen Evolution
Source: Adv Sci (Weinh). 2025 Mar 7;12(17):2416663. doi: 10.1002/advs.202416663 (PMC12061335; doi:10.1002/advs.202416663)
Supplement: Supplementary file 1 — Supporting Information [file ADVS-12-2416663-s001.pdf]

ADVANCED SCIENCE
Open Access

Supporting Information

for *Adv. Sci.*, DOI 10.1002/advs.202416663

Modulating Iron Crystals with Lattice Chalcophile-Siderophile Elements for Selective Dechlorinations Over Hydrogen Evolution

*Xiaohong Hu, Qianhai Zhou, Du Chen, Zhongyuan Guo, Yiman Gao, Chaohuang Chen, Jie Hou, Vincent Noël, Daohui Lin, Lizhong Zhu and Jiang Xu**

Supporting Information
©Wiley-VCH 2019
69451 Weinheim, Germany

Modulating Iron Crystals with Lattice Chalcophile-Siderophile Elements for Selective Dechlorinations over Hydrogen Evolution

*Xiaohong Hu[1], Qianhai Zhou[1], Du Chen[1], Zhongyuan Guo[1], Yiman Gao[1], Chaohuang Chen[1], Jie Hou[1,2], Vincent Noël[3], Daohui Lin[1,2], Lizhong Zhu[1,2], Jiang Xu[1,2]**

X. H. Hu, Q. H. Zhou, D. Chen, Z. Y. Guo, Y. M. Gao, C. H. Chen, J. Hou, D. H. Lin, L. Z. Zhu, J. Xu

College of Environmental and Resource Sciences

Zhejiang University

Hangzhou, 310058, China

E-mail: xujiang6@zju.edu.cn

J. Hou, D. H. Lin, L. Z. Zhu, J. Xu

Zhejiang Provincial Key Laboratory of Organic Pollution Process and Control

Zhejiang University

Hangzhou, 310058, China

V. Noël

Stanford Synchrotron Radiation Lightsource

SLAC National Accelerator Laboratory

Menlo Park, CA, 94025, USA

SUPPORTING INFORMATION

1. Table of Contents

2. Experimental Procedures 3
   Chemicals and Reagents 3
   Synthesis of Materials 3
   Structural Characterizations 3
   Physicochemical Property Analysis 5
   Calculation of Charge Density Distribution and *H Adsorption Energy 5
   Batch Experiments 6
   Reduction Products Analysis Methods 6
   Reactivity and Selectivity Evaluation 6
   Environmental Friendliness and Techno-economic Assessment 7
3. Supplementary Figures 9
   Figure S1. XRD patterns of lattice Co and/or S doped nFe^0 materials 9
   Figure S2. Elemental distribution and elemental co-associations in the line scans of S-nFe^0 materials 10
   Figure S3. Morphology, surface area, and Fe^0 content of nFe^0 and lattice-doped nFe^0 11
   Figure S4. The measured Co/Fe, and Cu/Fe molar ratio of lattice-doped nFe^0 materials 12
   Figure S5. The measured S/Fe molar ratio of lattice-doped nFe^0 materials 13
   Figure S6. Shell fitting of lattice Cu and S-doped nFe^0 materials 14
   Figure S7. Fe 2p XPS analysis of lattice S and/or Cu doped nFe^0 materials 15
   Figure S8. The proposed structures of lattice S and/or Cu doped nFe^0 materials 16
   Figure S9. Nyquist plots of lattice S and/or Cu doped nFe^0 materials 17
   Figure S10. Accumulated H2 of lattice S and/or Cu doped nFe^0 materials 18
   Figure S11. Comparison of TCE and FF removal activity before and after 120-day stability tests 19
   Figure S12. Carbon fractions, accumulated H_2, and electron efficiency during TCE removal. 20
   Figure S13. Concentrations of FF and its degradation products, accumulated H_2, and electron efficiency 21
   Figure S14. Leached ratio of Cu, Fe, and S elements in Cu-S-nFe^0 particles after 7 d/30 d of FF reaction 22
   Figure S15. Linear correlations between resistance and K_{SA}, HER, and EE 23
   Figure S16. Linear correlations between β_a and K_{SA}, HER, and EE 24
   Figure S17. Linear correlations between β_c and K_{SA}, HER, and EE 25
   Figure S18. Linear correlations between WCA and K_{SA}, HER, and EE 26
   Figure S19. Carbon fractions, accumulated H_2, and electron efficiency during TCE removal in real GW 27
   Figure S20. Concentrations of FF and its degradation products, accumulated H_2, and electron efficiency 28
   Figure S21. Leached ratio of Cu, Fe, and S elements in Cu-S-nFe^0 particles after 20 d of TCE reaction 29
   Scheme S1. Process flow for calculating environmental indices for inputs and outputs 30
4. Supplementary Tables 31
   Table S1. Results of the shell fits of Fe K-edge EXAFS 31
   Table S2. General Effect Index (GEI) calculated for the synthesis of Cu-S-nFe^0 32
   Table S3. Material usage cost of different materials for 1 m^3 remediation 33
   Table S4. Properties of the groundwater sample 34
   Table S5. Input chemical and energy costs of 1 kg Cu-S-nFe^0 production 35
5. References 36
6. Author Contributions 37

2. Experimental Procedures

Chemicals and Reagents

$FeCl_3$ (≥99.9%) was purchased from Shanghai Aladdin Biochemical Technology Co., Ltd. $CuCl_2 \cdot 2H_2O$ (99.9% metals basis), $Na_2S_2O_4$ (≥90%) and formic acid (for HPLC, ⩾99%) were supplied by Shanghai Macklin Biochemical Co., Ltd. $NaBH_4$ (≥98%), HCl (37%), NaOH (≥96%), HNO_3 (65–68%), H_2SO_4 (95%–98%), and Methanol (for HPLC, ≥99.9%) were acquired from Sinopharm Group Chemical Reagent Co., Ltd. Trichloroethylene (TCE, >99.5%) and flufenicol (FF, 98%) were obtained from Aladdin Corporation (Shanghai, China). A mixture of standard gases (1000 ppm C_2H_2, C_2H_4, and C_2H_6) were purchased from Dalian Special Gases Co., Ltd. All chemicals were employed without any other purification. Deionized water (18.2 MΩ·cm, pH ≈ 6.5, measured by Mettler Toledo S213-Water) was acquired from a Milli-Q ultra-pure systemused and deoxygenated deionized water was obtained by N_2 (≥99.999%) purging for about 1 hour.

Synthesis of Materials

The nFe^0, $Cu\text{-}nFe^0$, $S\text{-}nFe^0$, and $Cu\text{-}S\text{-}nFe^0$ nanoparticles were synthesized according to the previously reported one-step methods.[1] In all cases, the concentration of Fe^{3+} was kept at 10 g L^{-1}. The dosed Cu/Fe molar ratio of $Cu\text{-}nFe^0$ and $Cu\text{-}S\text{-}nFe^0$ was 0.01. The dosed S/Fe molar ratio of $S\text{-}nFe^0$ and $Cu\text{-}S\text{-}nFe^0$ was 0.14. Typically, the $Cu\text{-}S\text{-}nFe^0$ particles were synthesized by dropwise addition (~7 mL min^{-1}) of 200 mL of 34 g L^{-1} $NaBH_4$ and 2.2 g L^{-1} $Na_2S_2O_4$ solution into a continuously stirred (400 rpm) and N_2-purged 200 mL of Cu^{2+} and Fe^{3+} solution. The resulting suspensions were washed three times with deoxygenated deionized water and then vacuum dried at 60 °C overnight. After 2 hours of stabilization, during which air was slowly released into the vacuum, the dry particles were transferred to an anaerobic glovebox. The particles were then ground into a powder using an agate mortar, and the resulting powder was stored in sealed vials prior to use. The other materials (nFe^0, $Cu\text{-}nFe^0$, and $S\text{-}nFe^0$) were synthesized in the same manner, with the only differences being the absence of Cu^{2+} and/or $Na_2S_2O_4$. The synthesis methods for $Co\text{-}nFe^0$ and $Co\text{-}S\text{-}nFe^0$ are the same as those used for $Cu\text{-}nFe^0$ and $Cu\text{-}S\text{-}nFe^0$, with the only difference being the substitution of Cu^{2+} with Co^{2+} (using $CoCl_2 \cdot 6H_2O$).

Structural Characterizations

The Fe^0 content, Cu/Fe and S/Fe molar ratios in the particles ($[Cu/Fe]_{measured}$ and $[S/Fe]_{measured}$) were determined as the same as previous studies.[1b] Specifically, the total Cu, S, and Fe contents in the final $Cu\text{-}S\text{-}nFe^0$ material were measured by Inductively Coupled Plasma-Optical Emission Spectroscopy (ICP-OES, Agilent 5110), after digesting approximately 10 mg of the material with 10 mL of aqua regia. The molar ratios of Cu and S were then calculated based on the measured elemental contents. The

specific surface area of the samples was determined via Brunauer–Emmett–Teller (BET, Quantachrome 02108-KR-1). The crystal structures, surface species, morphologies, and elemental distributions of the samples were determined via X-ray diffraction (XRD, Bruker D8 Advance, Germany) using a Cu Kα X-ray source, X-ray Photoelectron Spectroscopy (XPS, ESCALAB 250Xi, Thermo Fisher Scientific, USA), and High-Angle Annular Dark-Field Scanning Transmission Electron Microscopy (HAADF-STEM, Talos F200X, Thermo Scientific, USA) equipped with energy dispersive X-ray system (EDS, FEI Super X, USA), respectively. The coordination environment of central Fe was determined by examining features recognition of Extended X-ray Absorption Fine Structure (EXAFS) at the Fe K-edge, along with relative shell fitting of Fourier transformed spectra and wavelet transform analysis via a bench-top synchrotron radiation system (RapidXAFS, Anhui, China). The distributions of Cu and Fe elements based on single particles were measured by spICP-TOF-MS (TOFWerk, Zurich, Switzerland).

spICP-TOF-MS analysis. The elemental compositions of single particles were measured by spICP-TOF-MS (TOFWerk, Zurich, Switzerland). This method allows for the simultaneous detection of all elements in the sample, determination of the relative ratios of the main elements in each particle, and multi-element analysis of individual particles. The limits of detection were calculated according to IUPAC for low signals (LOD (counts) = 3.29σ blank (counts) + 2.71). Any integration time point above this threshold is considered a single particle event. An integration time of 20 ms was used, and the Poisson distribution was applied for analysis. The transport efficiency was determined using a 50 nm Au nanoparticles suspension (Sigma Aldrich). The instrument was operated in KED collision mode, utilizing He gas collision to reduce ArO^+ interference. For the measurement of Fe or Cu elements, calibration was performed using mixed standards containing 48 elements (10 ppm, ICP-MS-68A-A-100, 2% HNO_3), covering a range from 0.1 ppb to 10 ppb in deionized water. Before the test, an appropriate amount of sample powder was mixed with 10 mL of chromatographically pure absolute ethanol to create a stock solution, followed by ultrasonic dispersion. It was only at the time of the test that 10 μL of this stock solution was transferred into 10 mL of deoxygenated deionized water, resulting in a dilution factor of 10^3–10^6 times. This diluted solution was then quickly sonicated and analyzed on the instrument.

X-ray absorption fine structure (XAFS) spectroscopy analyses. The XAS spectroscopy was carried out using the Rapid XAFS 1M (Anhui Absorption Spectroscopy Analysis Instrument Co., Ltd.) by transmission mode and the Ge (620) spherically bent crystal analyzer was used for Fe K-edge. For Fe K-edge spectra, a mass of sample calculated by a software was diluted in micron-sized boron nitride and thoroughly mixed by grinding. A disk with a diameter of 13 mm can be get after pressing by ~2 tons of pressure. After sealing with Scotch® Magic™ tape and securing to the sample tray, the sample disk tested immediately. The spectra were energy calibrated using an iron metal foil with an energy setting of 7112 eV. Before shell fitting analysis with Artemis software, data was calibrated by Athena software including background deduction, E_0 determination, parallel sample data integration (merge), and outlier removal (deglitch and truncate data).[2] Metallic iron (Fe^0, space group Lm-3m; a = b = c = 2.850 Å and

α = β = γ = 90°),[3] FeS_2 (Pyrite, space group Pa3, a = b = c = 5.404 Å and α = β = γ = 90°),[3] FeS (Mackinawite, space group P4/nmm:1, a = b = 3.673 Å, c = 5.033 Å and α = β = γ = 90°),[4] Cu doped metallic iron ($CuFe^0$, space group Im-3m, a = b = c = 2.8678 Å and α = β = γ = 90°),[5] and $CuFeS_2$ (Chalcopyrite, space group F-43m, a = b = c = 5.228 Å and α = β = γ = 90°)[6] structural parameters were used to run Feff and fit paths for shell fitting analysis of samples. The fitting was taken in the R space and the results are displayed in Figure S6. Combining all the fitting parameters as well as other experimental data in this paper, the best path fitting results and its crystal parameters are displayed in Figure 2d−f, and Table S1.

Physicochemical Property Analysis

Electrochemical analyses. Refer to literature basis,[3] Tafel curves and EIS were performed with the CHI-660 electrochemical workstation (CH Instruments, Chenhua, Shanghai, China) with a standard three-electrode system using 10 mM NaCl as electrolyte. After weighing ~100 mg of material (nFe^0, Cu-nFe^0, S-nFe^0, or Cu-S-nFe^0), use a hydraulic press to compress it onto a 0.8 cm × 1.2 cm titanium mesh. A 0.8 cm × 0.8 cm square piece of material will form on the titanium mesh under a force of ~2 tons within a customized HF-2Y model (Tianjin Tiangong Optical Instrument Co., Ltd.). Before electrochemical characterization, the electrolyte solution (10 mM NaCl) was deoxidation by filling with N_2. After that, the open circuit potential (OCP) measurements, electrochemical impedance spectroscopy (EIS), and Tafel analysis were testing in sequence, with nFe^0, Cu-nFe^0, S-nFe^0, or Cu-S-nFe^0 electrode as the working electrode, a platinum wire (Sigma-Aldrich) as the counter electrode, and an Ag/AgCl (saturated KCl) as the reference electrode. The EIS was carried out after recording OCP for 1 h with a 5 mV amplitude sinusoidal potential perturbation over a frequency range of 10000 Hz to 1000000 Hz. Finally, Tafel analysis was conducted at a scan rate of 10 mV s^{-1} and ±250 mV relative to OCP. Throughout the process, the electrolyte solution was maintained under a N_2 atmosphere to keep deoxygenation.

To assess the hydrophobicity, a 0.6-cm diameter disk was fashioned from approximately 200 mg of material using the hydraulic press, followed by overnight drying at 60 °C in a vacuum drier. The subsequent water contact angle measurement (WCA) was conducted on the OSA200 Optical surface analyzer from Ningbo NB Scientific Instrument Co., Ltd.

The longevity of different materials in water was evaluated by monitoring the loss of Fe^0 content based on hydrogen evolution ($Fe^0 + 2H_2O \rightarrow Fe^{2+} + 2OH^- + H_2$).[7] This approach is based on prior studies that have used hydrogen evolution as an indicator of Fe^0-based materials' longevity.[1b, 7] More specifically, the maximum hydrogen evolution is first calculated based on the Fe^0 content (1 mol of Fe^0 → 1 mol of H_2). The hydrogen accumulated during the rapid consumption phase is then subtracted from this maximum value. The remaining hydrogen production (Fe^0 content) is divided by the steady-state hydrogen evolution rate to estimate the duration of hydrogen evolution. The final longevity is obtained by adding the time from the rapid consumption phase to the steady-state phase, giving the total time in

days. Through changes in hydrophobicity and Fe^0 content as indicators to evaluate the stability of materials in the atmospheric environment.

Calculation of Charge Density Distribution and *H Adsorption Energy

All spin-polarized calculations have been performed within density functional theory (DFT, VASP code) with the RPBE functional for electron exchange and correlation.[8] The Kohn-Sham equations are solved using a plane wave basis with a cutoff of 520 eV.[9] The van der Waals interaction is described by the DFT-D3 scheme.[10] The convergence tolerance for the energy on each atom and residual force have been set to 10^{-5} eV and 0.02 eV/Å. The Brillouin zone has been sampled with the Monkhorst-Pack mesh with a K-point set of 3×3×1 for all supercells in reciprocal space.[11] To investigate the impact of lattice S and Cu dopants on hydrogen (*H) adsorption, pure iron (nFe^0), Cu-doped iron (Cu-nFe^0), S-doped iron (S-nFe^0), and Cu-S co-doped iron (Cu-S-nFe^0) models, have been built with a 4-atom-layer thickness. During the geometric optimization, the bottom two layers have been constrained in the bulk positions. The vacuum layer in the *z*-direction is set to 15 Å, enough to prevent the interaction between periodical images. These atomic models are consisted of 48 atoms, with the [S/Fe] and [Cu/Fe] ratios close to the as-synthesized samples. The charge distribution has been analyzed by the Bader charge. The H adsorption energy ($\Delta E_{ads,\ H}$) has been defined as: $\Delta E_{ads,\ H} = E_{*H} - E_{slab} - 0.5*E_{H2}$, where E_{*H}, E_{slab}, E_{H2} represent electronic energies of the adsorbed *H, bare slab, and H_2, respectively. The more positive $\Delta E_{ads,\ H}$, the less likely it is for hydrogen to be adsorbed onto nFe^0-based materials.

Batch Experiments

Batch reactivity studies were referenced to previous studies.[12] The original concentration of TCE and FF was 70 µM and 0.15 mM, respectively. The dosage of materials was 1 g L^{-1} and the temperature was ~25 °C. Teflon Mininert valves were used to seal the serum vials, which were then placed on an end-over-end rotator set at a speed of 50 rpm for agitation. The degradations of TCE and FF were measured over 7 d and 6 h, respectively.

To assess environmentally relevant conditions, deoxygenated real groundwater (from a well in Hangzhou, Zhejiang Province, China) and deoxygenated deionized water were used to evaluate the reactivity and selectivity of these different materials. The corresponding physicochemical parameters of the real groundwater are summarized in Table S4. Reaction conditions were the same as described above.

Reduction Products Analysis Methods

A gas chromatography-flame ionization detector system (GC-FID, FuLi GC9790plus) was used to analyze TCE and its degradation products, where was equipped with a GS-Q column (0.53 mm × 30 m) (Agilent Technologies, USA). The aqueous concentrations of TCE dechlorination products were

calculated using their Henry's law constants.[1b] The Hydrogen evolution (H_2 gas) was monitored using a gas chromatography-thermal conductivity detector system (GC-TCD, FuLi GC9790plus) with a 2 m stainless-steel column (TDX-01). High-performance liquid chromatography (HPLC, Shimadzu, Japan) was utilized to determine the concentrations of FF and its main dechlorinated products, namely deschloro FF (DE) and dideschloro FF (DI), using a BDS Hypersil™ C18 column (Thermo Scientific, dimensions 150 × 4.6 mm, particle size 5 μm). The mobile phase consisted of a mixture of methanol and 0.05% formic acid solution in a ratio of 30:70 (v/v), with an injection volume of 10 μL.

Reactivity and Selectivity Evaluation

A preliminary assessment was conducted to evaluate the reactivity (i.e., K_{obs} and K_{SA}) and selectivity (i.e., electron efficiency (EE)) of the materials in accordance with previously established methodologies.[1b, 3, 7] The K_{obs} values are expressed as pseudo-first-order rate constants for the dechlorination of TCE or FF, and the K_{SA} represents the K_{obs} normalized per unit surface area and material dosage. The electron efficiency is interpreted as the electron consumption for the reduction of the target contaminant (TCE or FF) relative to the total electron consumption of all reductants in the system, including H_2O.

Dechlorination enhancement and Hydrogen evolution (HER) inhibition were based on a comparison between the doped materials (Cu-nFe^0, S-nFe^0, and Cu-S-nFe^0) and the nFe^0 system. Dechlorination enhancement was calculated as the fold of the improvement in K_{SA} in the doped material systems relative to that in the nFe^0 system, while HER inhibition was calculated as the fold of accumulated hydrogen in the nFe^0 system relative to that in the doped material systems, when >85% TCE or >95% FF degradation is achieved.

Statistical Analysis

All experimental data are presented as the mean of three independent parallel experiments. The sample standard deviation (SD) of the dataset was calculated using the STDEV.S function in Microsoft Excel. Linear fitting and graph plotting were performed using the software Origin.

Environmental Friendliness and Techno-economic Assessment

Techno-economic analysis was performed for calculating the cost of the synthesis of Cu-S-nFe^0 (i.e., material cost) and the process of remediation.[13] The material cost consists of input reagents and energy consumption costs (electricity and water). It is important to note that reagent prices fluctuate in real time; the calculations are based on the prices current at the time of analysis (Table S5). Water and electricity expenses are determined based on the tariffs in effect for Hangzhou, Zhejiang Province (Table S5).

The General Effect Index (GEI), derived from the Biwer-Heinzle environmental evaluation framework, was utilized to ascertain environmental friendliness of the synthesis.[13-14] By analyzing the mass

indicators of the compounds engaged in the material synthesis process and categorizing them according to the A/B/C classification system within 14 primary ecological impact categories, the GEI is calculated. A lower GEI indicates a reduced environmental footprint.

The predictable remediation costs discussed in this research encompass material usage costs, drilling costs, monitoring costs, preservation costs, and the costs associated with installation and injection procedures.[13, 15] We assume a groundwater volume of 600 m^3, with a material dosage of 1 g L^{-1} and contamination concentrations of TCE at 70 μM and FF at 0.15 mM, consistent with laboratory experiment conditions. The quantities and expenditures for the respective materials—namely nFe^0, Cu-nFe^0, S-nFe^0, and Cu-S-nFe^0—are dependent on their electronic selectivity and the total mass of contaminant substances (TCE or FF) present in the groundwater. These values are detailed in Table S3. The material usage is calculated using the following equation:

$$\text{Material usage} = M_{\text{contaminant}} \div S_{\text{material}} \div R_{\text{contaminant}}$$

Where $M_{\text{contaminant}}$ is the total mass of contaminant substances (TCE or FF). S_{material} refers to the electronic selectivity of the various materials tested in groundwater. $R_{\text{contaminant}}$ represents the removal ratio of the different materials in decontaminating the specified pollutants (TCE or FF).

Consequently, the material usage cost incurred during the remediation process is determined by the product of the cost of material synthesis and the amount of material used.

The preservation costs is calculated with the following equation:

$$C_{\text{preservation}} = \eta \times C_{\text{synthesis}}$$

Where $C_{\text{preservation}}$ is the preservation costs, $C_{\text{synthesis}}$ is the material cost, η is the oxidation rate of materials stored in air.

The installation and injection costs refer to a document of U.S. Department of Defense (*Cost and Performance Report, ER-0221*), which is range from 38.94 $/$m^3$ to 393.40 $/$m^3$ accroding to the volume of groundwater. Thus, we took 192.29 $/$m^3$ as the injection costs based on volume of 600 m^3. The monitoring cost is refer to the EPA document (*Ambient Ground Water Quality Monitoring Cost Analysis*), including the groundwater sample collection costs and groundwater samlple analysis costs. The difference is that we assume there are four groundwater monitoring wells and two technicians. The drilling cost is refer to a average value (9180 $ per well) from a government document (*Well Drilling Costs*) of Central Valley Flood Protection Board of California.

3. Supplementary Figures

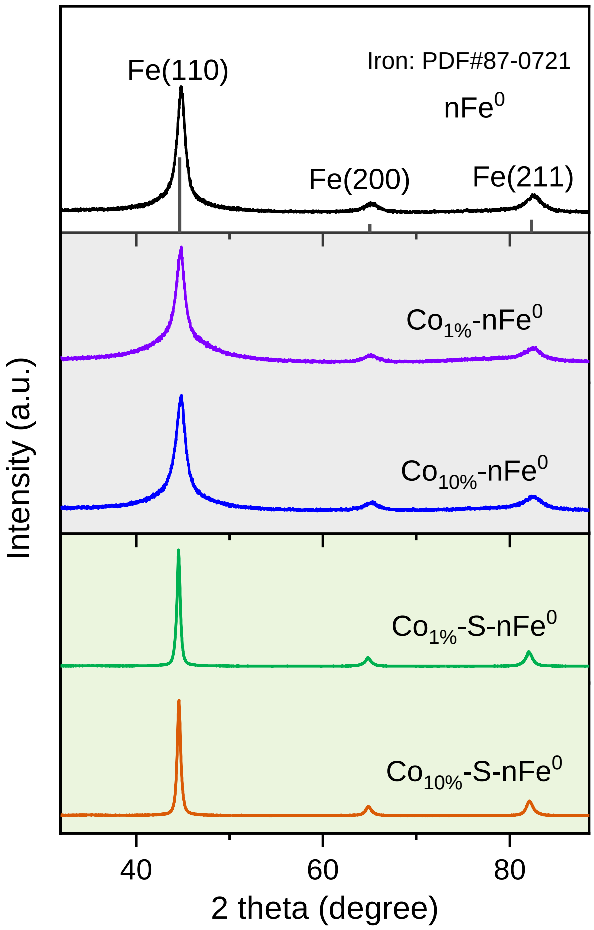

Figure S1. XRD patterns of lattice Co and/or S doped nFe^0 materials with the concentration of Co at 1% or 10% and $[S/Fe]_{dosed}$ = 0.14.

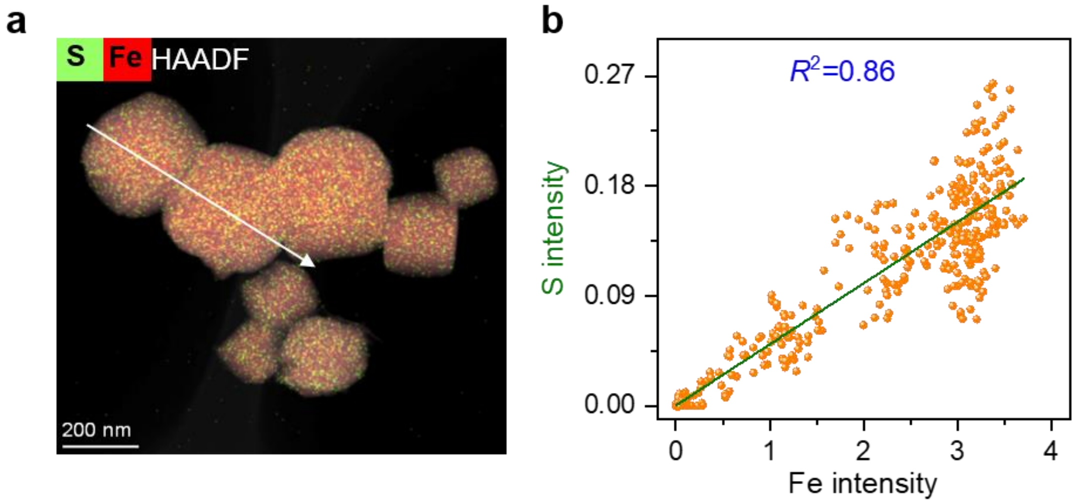

Figure S2. (a) Elemental distribution, and (b) elemental co-associations in the line scans of S-nFe^0 materials with $[S/Fe]_{dosed}$ = 0.14. The white arrow in (a) presents the scanning directions of the EDX line scanning. The green line in (b) means the fit line using the formula of y = a + bχ.

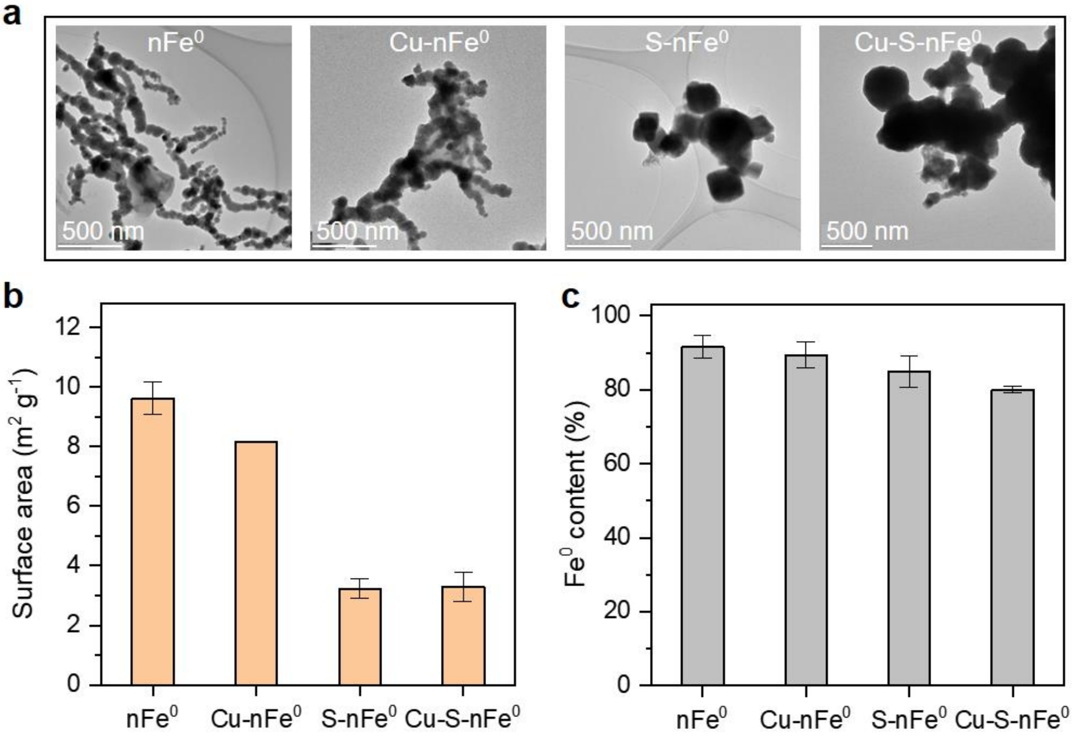

Figure S3. (a) Morphology, (b) surface area, and (c) Fe^0 content of nFe^0 and lattice-doped nFe^0 with 1% Cu or S ($[S/Fe]_{dosed}$=0.14). Data are presented as mean ± s.d. (n=3).

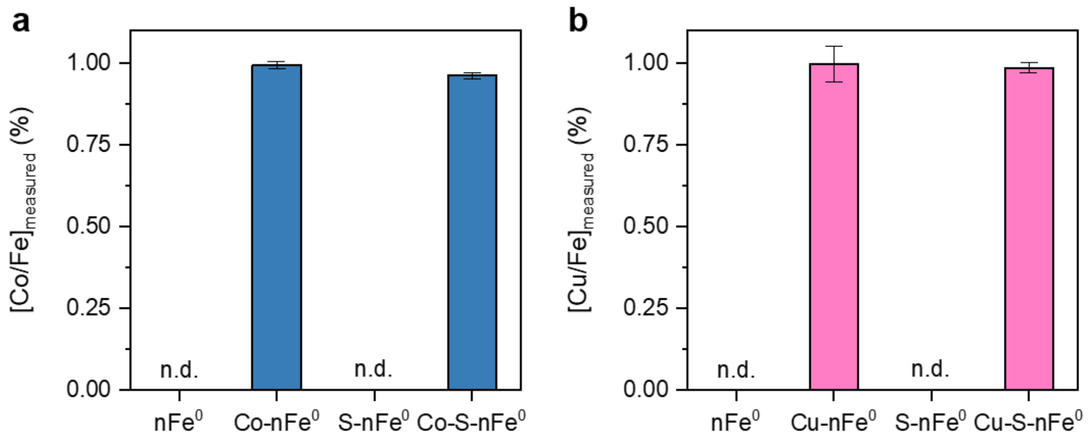

Figure S4. The measured (a) Co/Fe, and (b) Cu/Fe molar ratio of lattice-doped nFe^0 materials ($[Co/Fe]_{dosed}$=1%, $[Cu/Fe]_{dosed}$=1%, and $[S/Fe]_{dosed}$=0.14). Data are presented as mean ± s.d. (n=3). The n.d. means not detected.

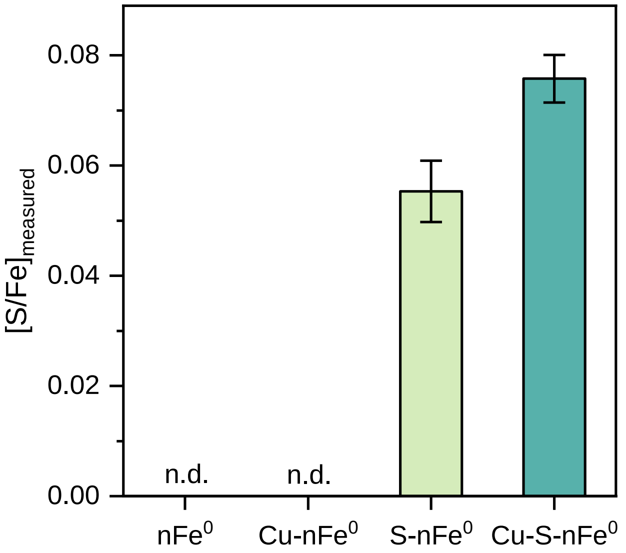

Figure S5. The measured S/Fe molar ratio of lattice-doped nFe^0 materials ($[Cu/Fe]_{dosed}$=1%, and $[S/Fe]_{dosed}$=0.14). Data are presented as mean ± s.d. (n=3). The abbreviation 'n.d.' is 'not detected'.

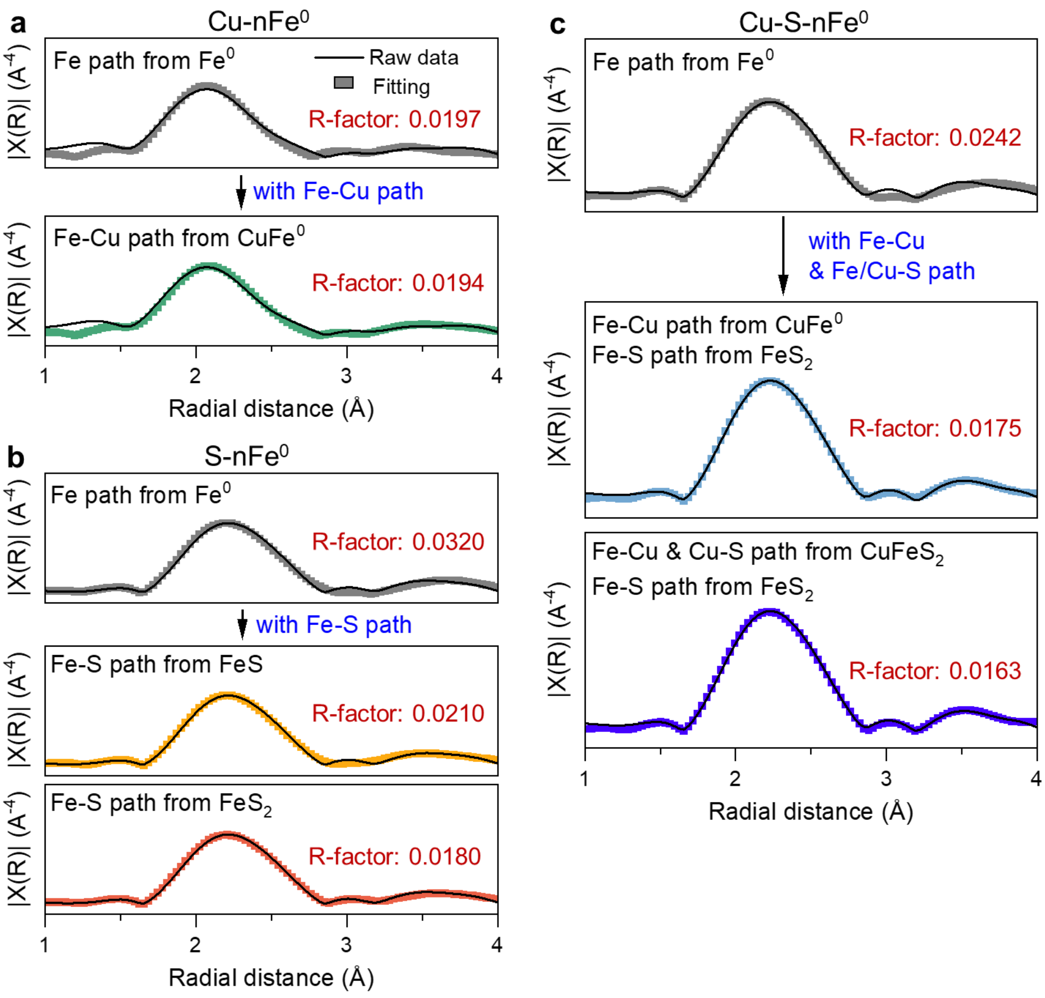

Figure S6. Shell fitting of lattice Cu and S-doped nFe^0 materials ($[Cu/Fe]_{dosed}$=1%, and $[S/Fe]_{dosed}$=0.14) using different paths from various models. R-factor reflects the satisfaction of the fitting result, with the lower value indicating better fit quality.

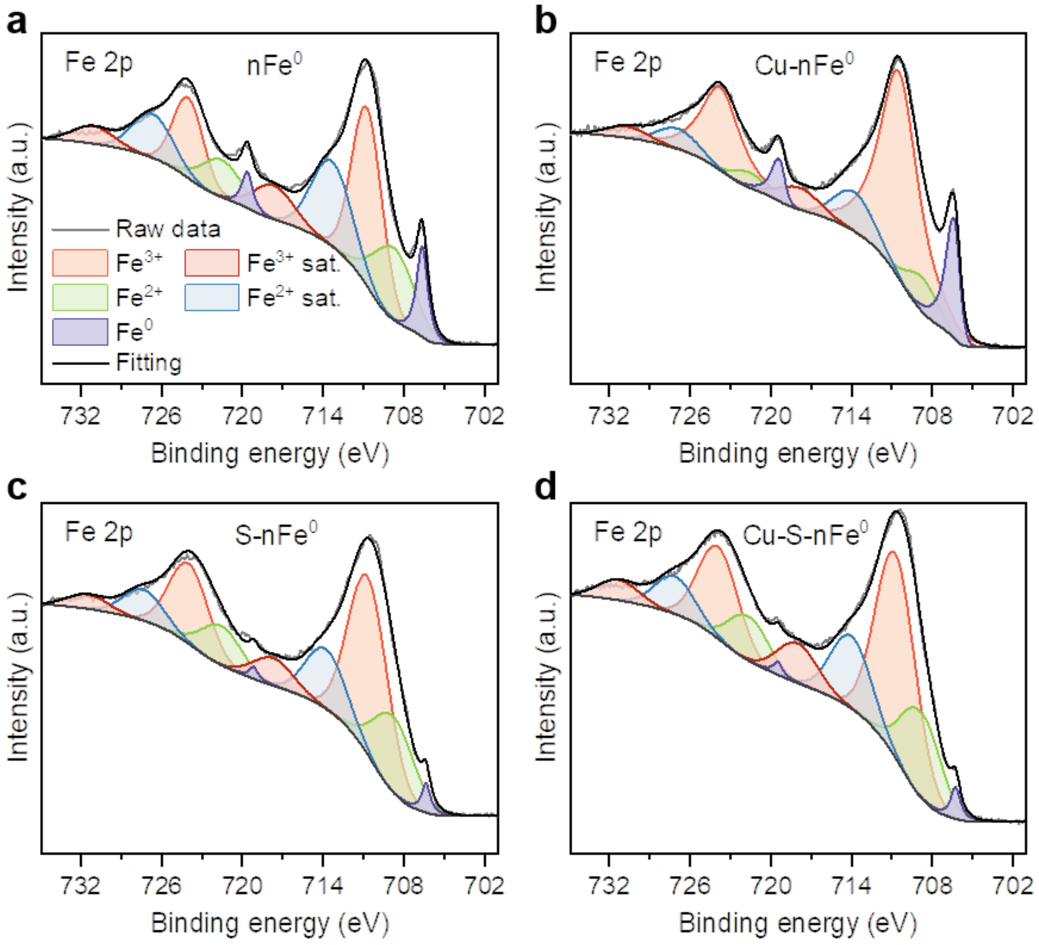

Figure S7. Fe 2p XPS analysis of lattice S and/or Cu doped nFe^0 materials ($[Cu/Fe]_{dosed}$=1%, and $[S/Fe]_{dosed}$=0.14). (a) nFe^0, (b) $Cu\text{-}nFe^0$, (c) $S\text{-}nFe^0$, and (d) $Cu\text{-}S\text{-}nFe^0$.

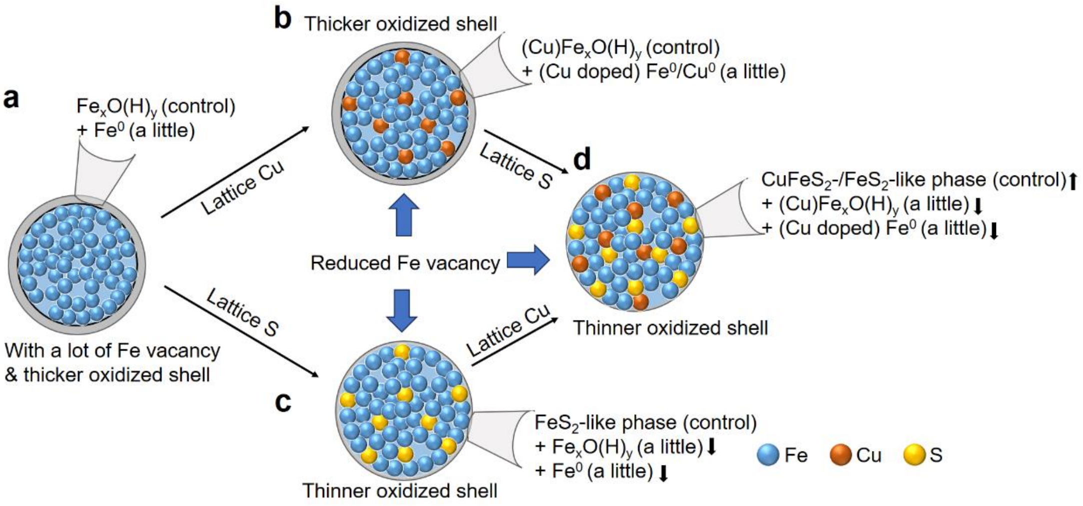

Figure S8. The proposed structures of lattice S and/or Cu doped nFe^0 materials ($[Cu/Fe]_{dosed}$=1%, and $[S/Fe]_{dosed}$=0.14): (a) nFe^0, (b) Cu-nFe^0, (c) S-nFe^0, and (d) Cu-S-nFe^0.

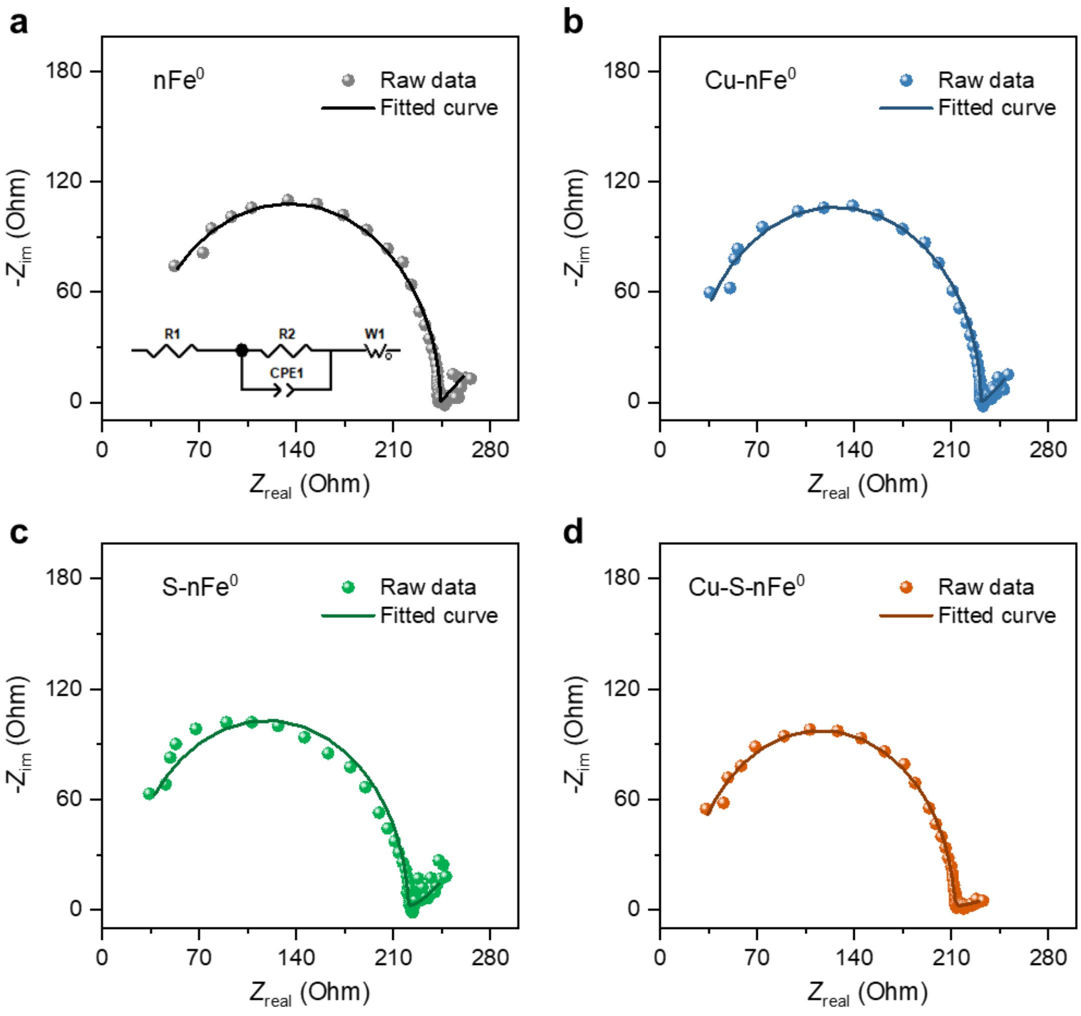

Figure S9. Nyquist plots of lattice S and/or Cu doped nFe^0 materials ($[Cu/Fe]_{dosed}$=1%, and $[S/Fe]_{dosed}$=0.14) and its fit to an equivalent circuit model comprised of electrolyte resistance (R1), charge transfer and contact resistance (R2), a constant phase element (CPE1), and a warburg element (open, W1).

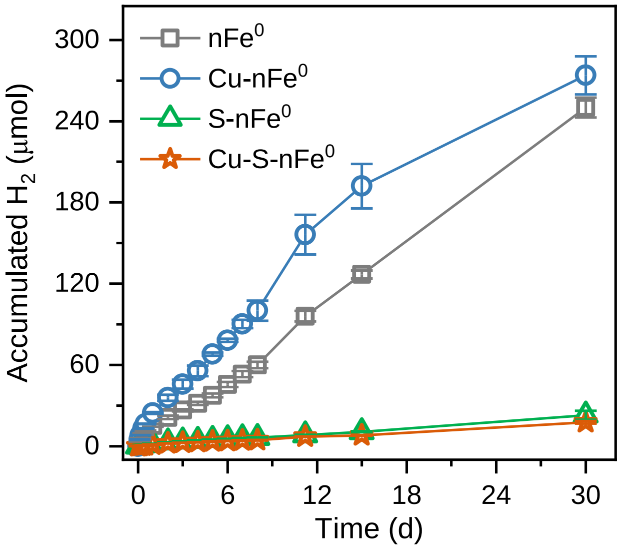

Figure S10. Accumulated H_2 of lattice S and/or Cu doped nFe^0 materials ($[Cu/Fe]_{dosed}$=1%, and $[S/Fe]_{dosed}$=0.14) in reaction with deoxygenated deionized water over time. Basic conditions are 1 g L^{-1} lattice-doped nFe^0 materials, pH ≈ 6.5, temperature ≈ 25 °C, and a speed of 50 rpm. Data are presented as mean ± s.d. (n=3).

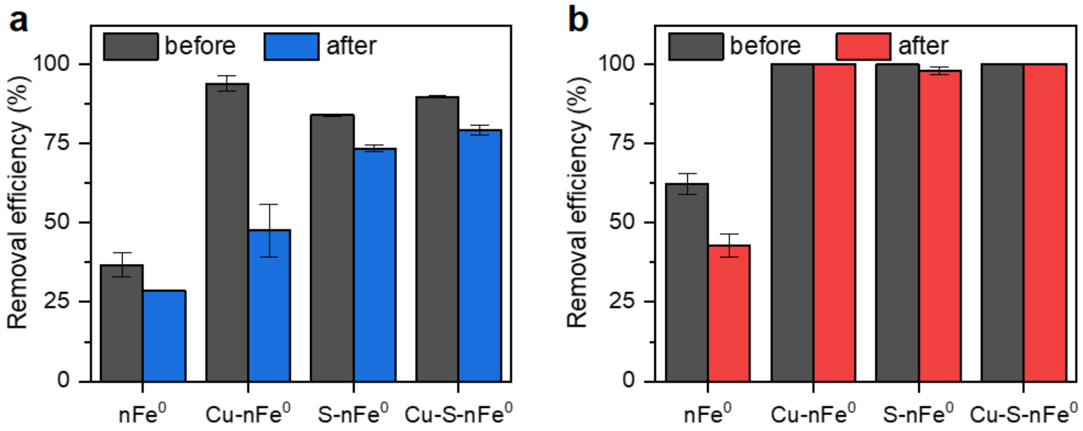

Figure S11. Comparison of (a) TCE and (b) FF removal efficiencies by materials being exposed in air for 120 days with that by fresh materials. Basic conditions are 1 g L^{-1} lattice-doped nFe^0 materials, $[Cu/Fe]_{dosed}$=1%, $[S/Fe]_{dosed}$=0.14, 70 µM TCE or 0.15 mM FF, pH ≈ 6.5, temperature ≈ 25 °C, reaction time=168 hours (TCE) and 6 hours (FF).

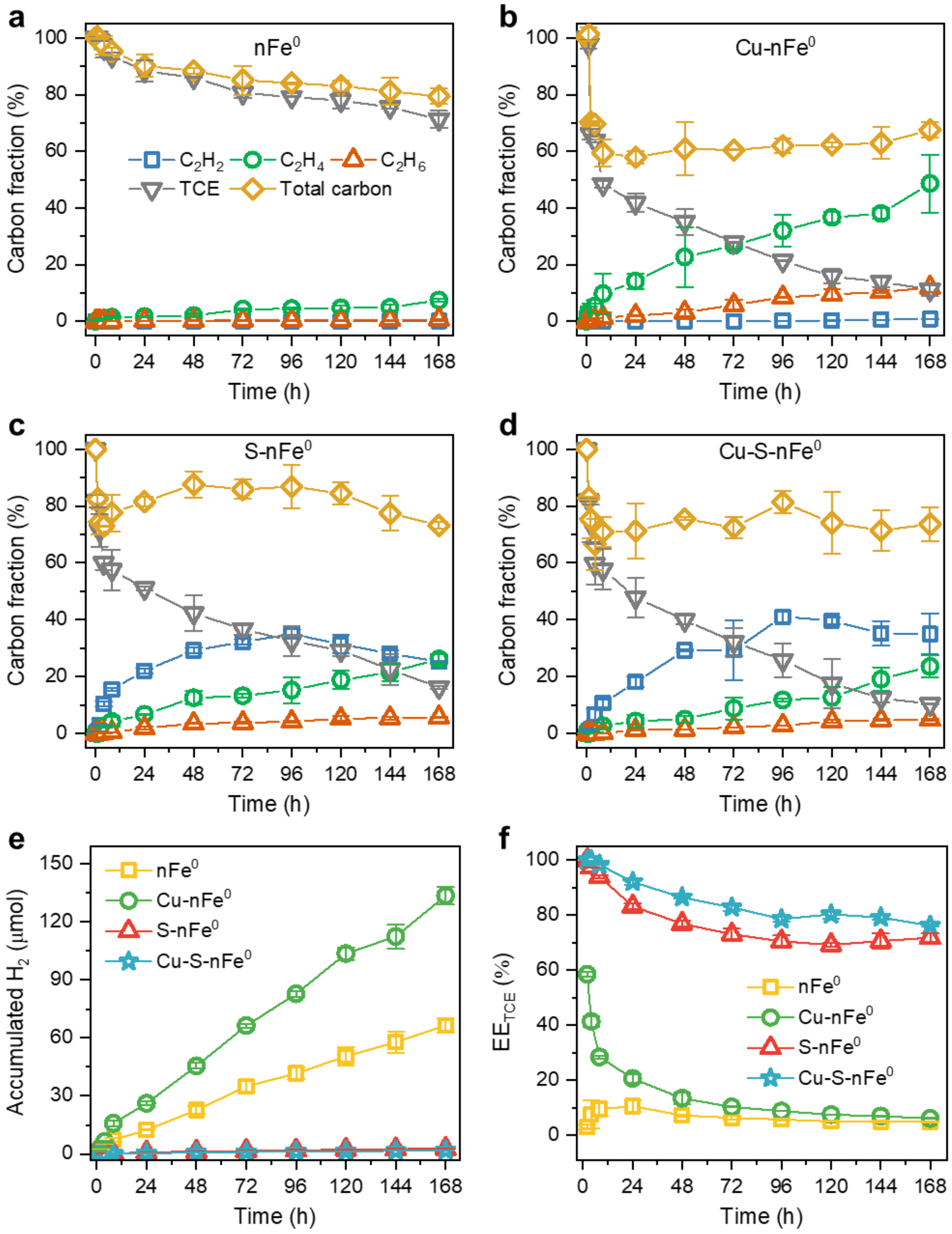

Figure S12. (a–d) Carbon fractions, (e) accumulated H2, and (f) electron efficiency (i.e., EE) during TCE removal by lattice S and/or Cu doped nFe^0 materials ($[Cu/Fe]_{dosed}$=1%, and $[S/Fe]_{dosed}$=0.14). Basic conditions are 1 g L^{-1} lattice-doped nFe^0 materials, 70 μM TCE, pH ≈ 6.5, temperature ≈ 25 °C, and a speed of 50 rpm. Data are presented as mean ± s.d. (n=3).

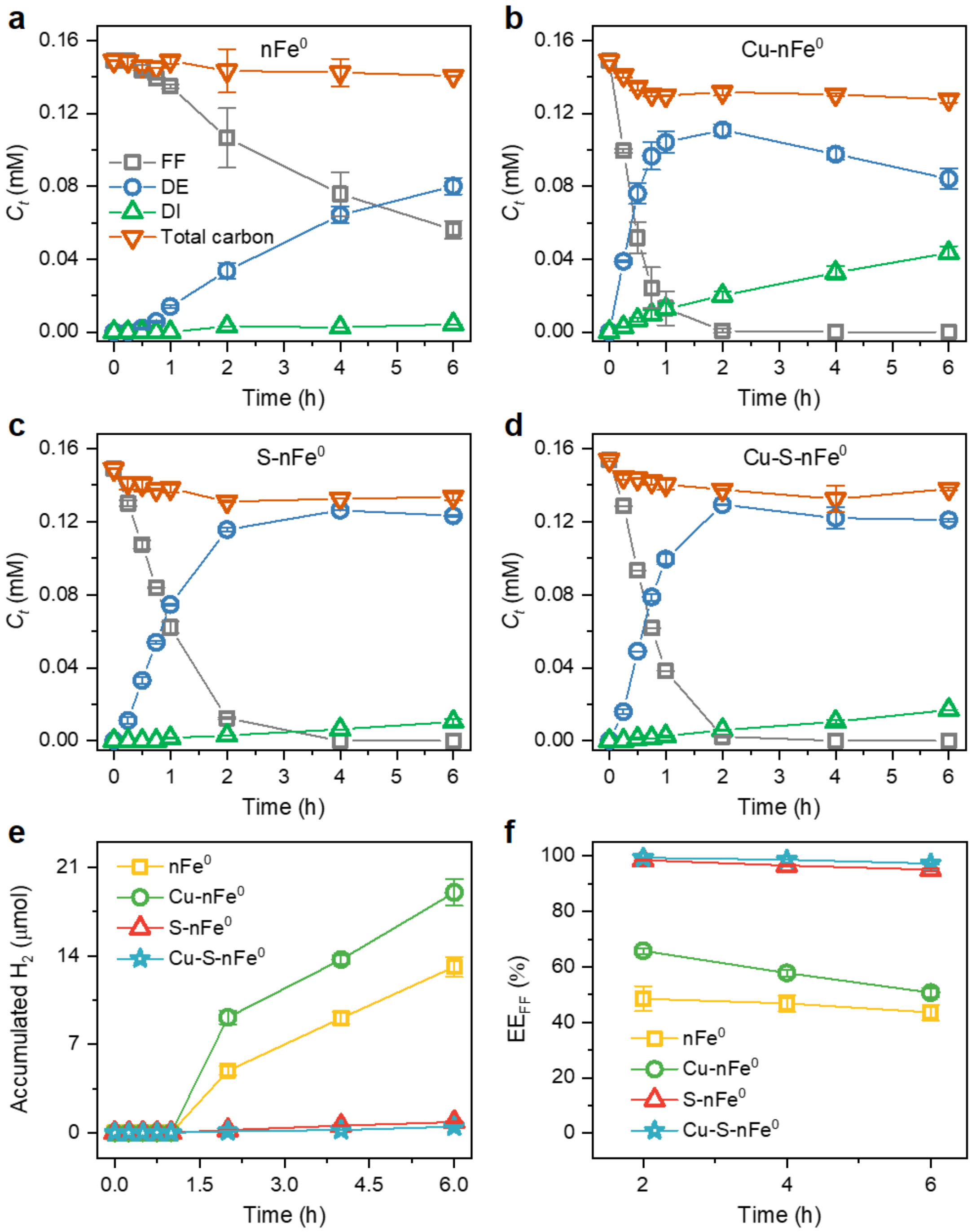

Figure S13. (a–d) Concentrations of FF and its degradation products, (e) accumulated H2, and (f) electron efficiency (i.e., EE) during FF removal by lattice S and/or Cu doped nFe^0 materials ($[Cu/Fe]_{dosed}$=1%, and $[S/Fe]_{dosed}$=0.14). The abbreviation 'DE' and 'DI' is 'deschloro FF' and 'dideschloro FF', respectively. Basic conditions are 1 g L^{-1} lattice-doped nFe^0 materials, 0.15 mM FF, pH ≈ 6.5, temperature ≈ 25 °C, and a speed of 50 rpm. Data are presented as mean ± s.d. (n=3).

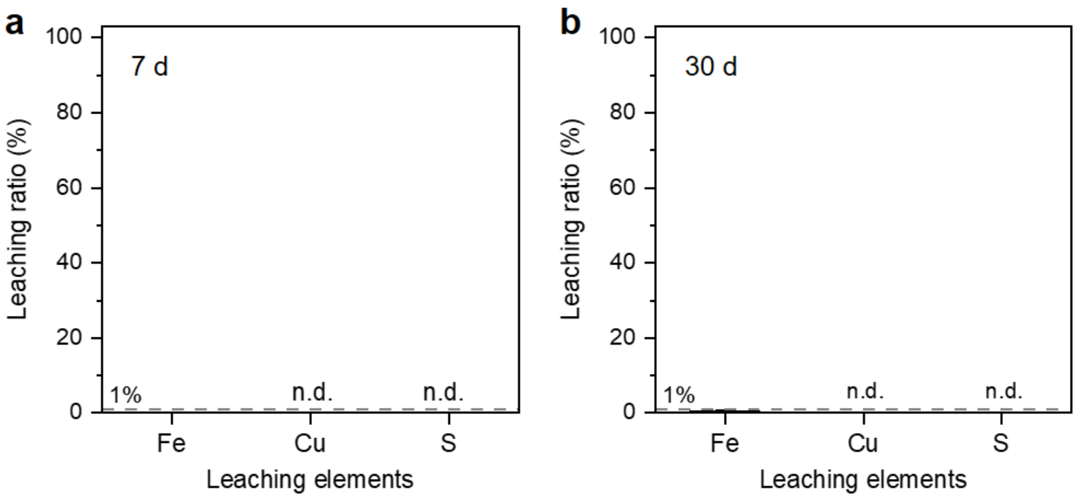

Figure S14. Leached ratio of Cu, Fe, and S elements in Cu-S-nFe^0 particles ($[Cu/Fe]_{dosed}$=1%, and $[S/Fe]_{dosed}$=0.14) after (a) 7 days and (b) 30 days of FF reaction. The abbreviation 'n.d.' is 'not detected'. Basic conditions are 1 g L^{-1} lattice-doped nFe^0 materials, 0.15 mM FF, pH ≈ 6.5, temperature ≈ 25 °C, and a speed of 50 rpm. Data are presented as mean ± s.d. (n=3).

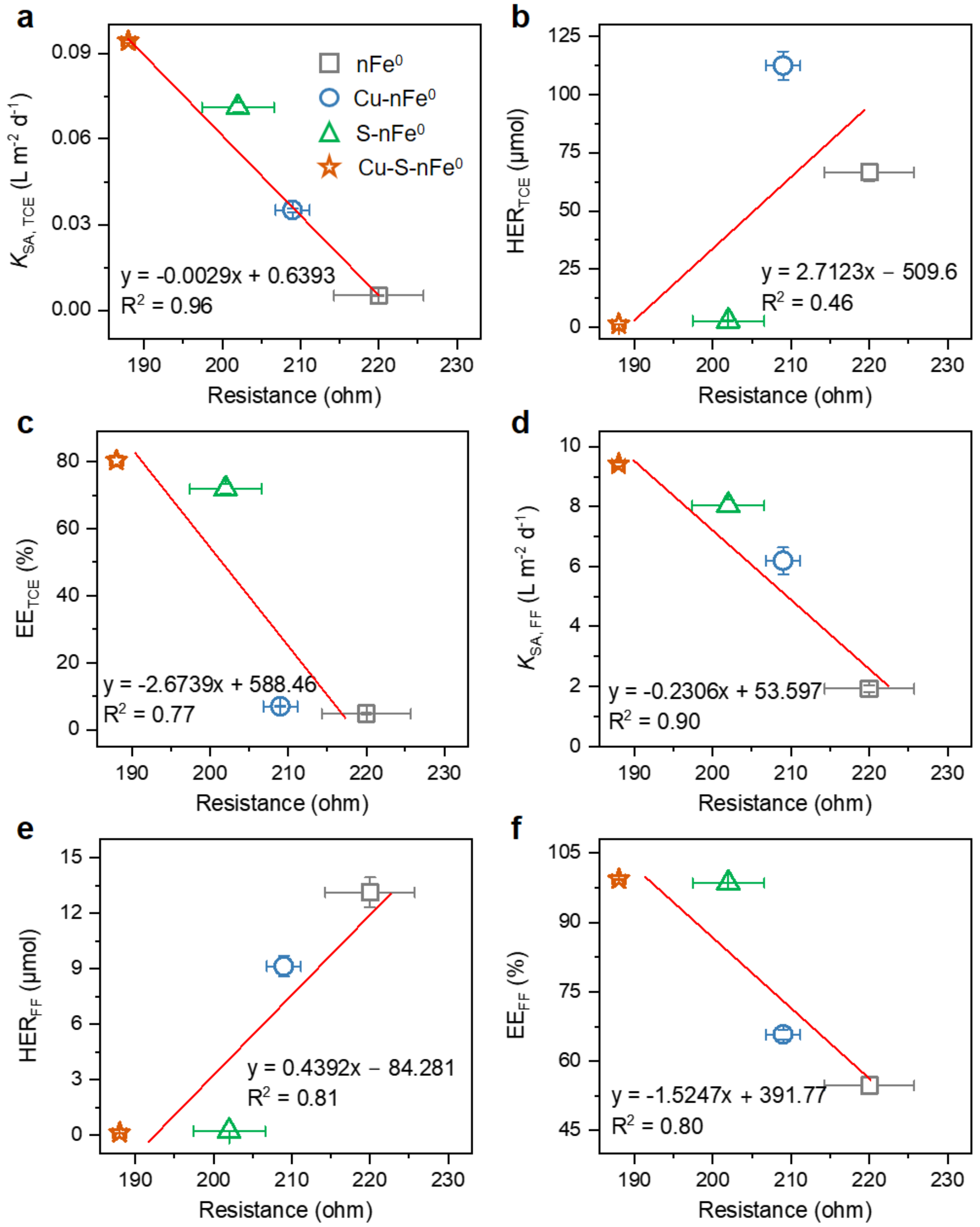

Figure S15. Linear correlations between resistance and dechlorination reactivity (K_{SA}), H_2 evolution (HER), and electron efficiency (EE) in lattice S and/or Cu doped nFe^0 materials ([Cu/Fe]$_{dosed}$=1%, and [S/Fe]$_{dosed}$=0.14) within (a–c) TCE systems and (e–f) FF systems.

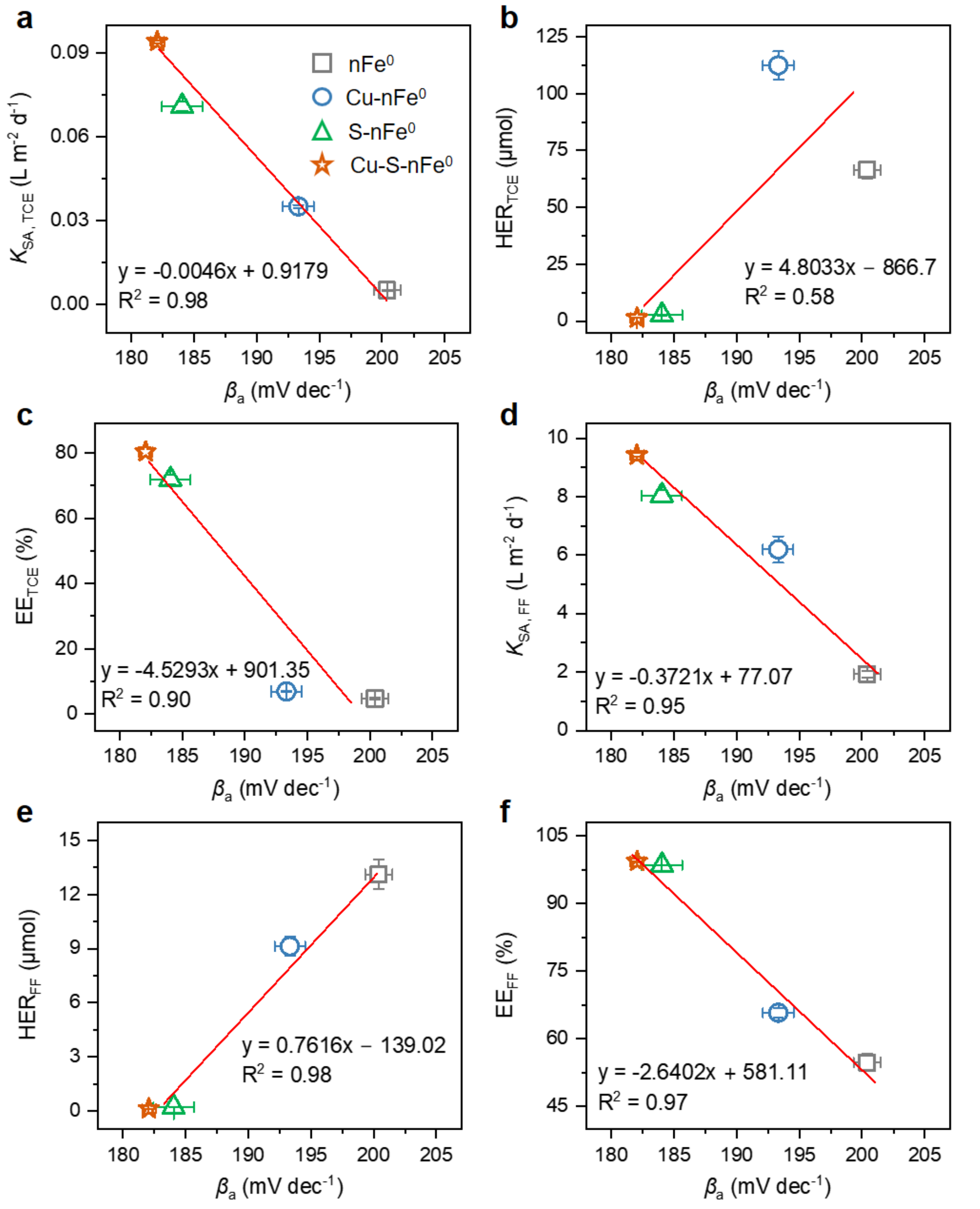

Figure S16. Linear correlations between β_a and dechlorination reactivity (K_{SA}), H_2 evolution (HER), and electron efficiency (EE) in lattice S and/or Cu doped nFe^0 materials ($[Cu/Fe]_{dosed}$=1%, and $[S/Fe]_{dosed}$=0.14) within (a–c) TCE systems and (e–f) FF systems.

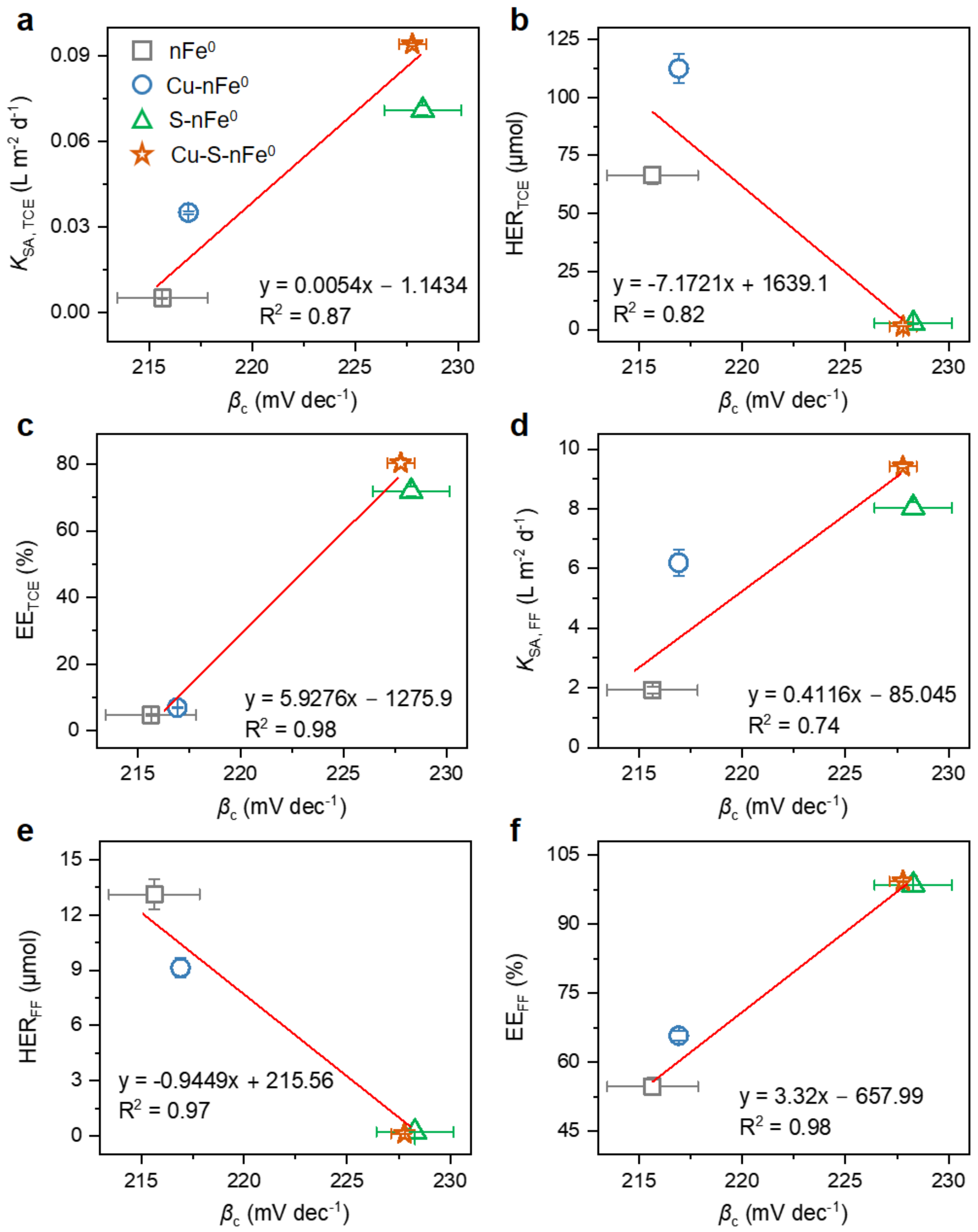

Figure S17. Linear correlations between β_c and dechlorination reactivity (K_{SA}), H_2 evolution (HER), and electron efficiency (EE) in lattice S and/or Cu doped nFe^0 materials ($[Cu/Fe]_{dosed}$=1%, and $[S/Fe]_{dosed}$=0.14) within (a–c) TCE systems and (e–f) FF systems.

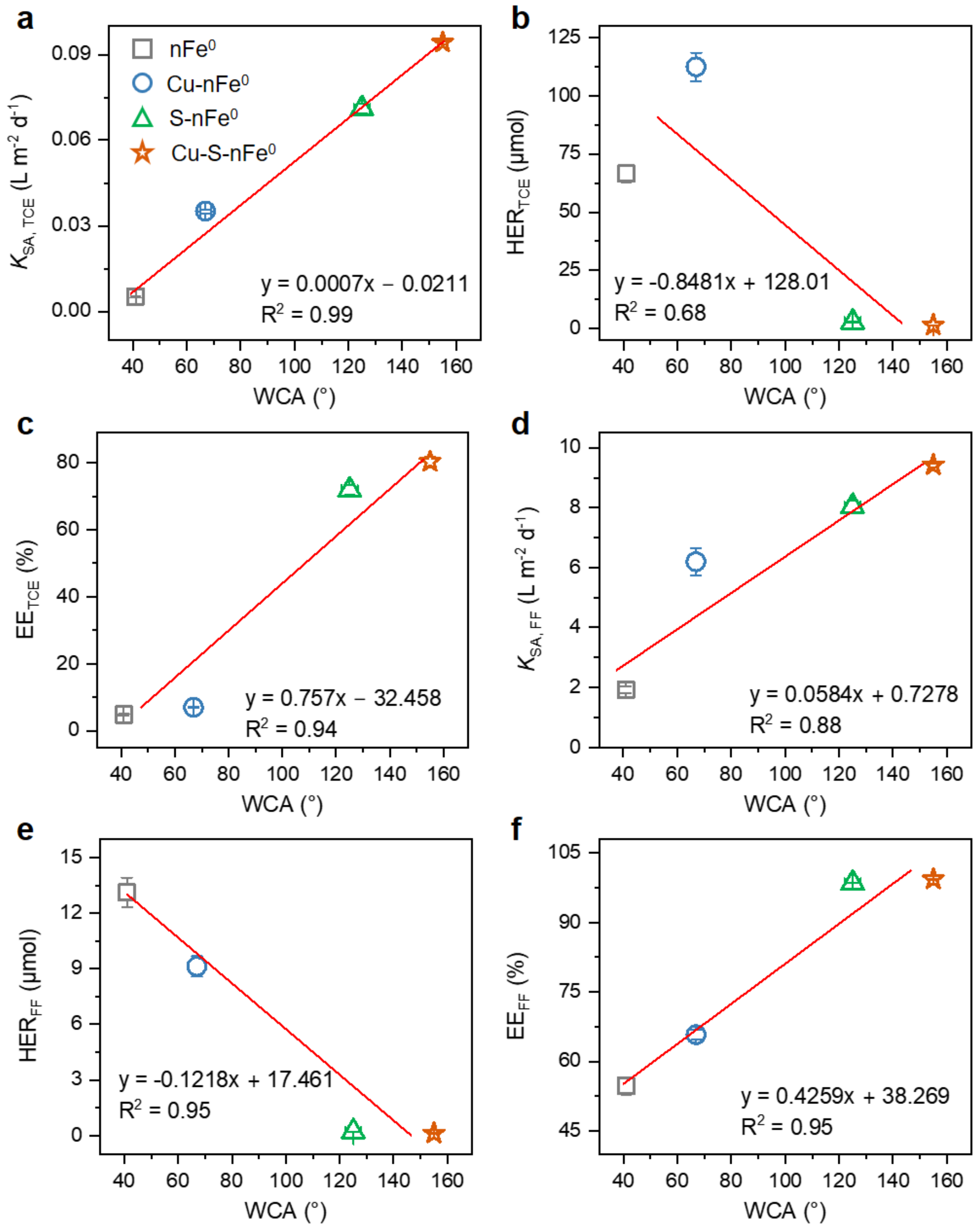

1

2 **Figure S18.** Linear correlations between water contact angle (WCA) and dechlorination reactivity (K_{SA}),
3 H_2 evolution (HER), and electron efficiency (EE) in lattice S and/or Cu doped nFe^0 materials
4 ($[Cu/Fe]_{dosed}$=1%, and $[S/Fe]_{dosed}$=0.14) within (a–c) TCE systems and (e–f) FF systems.

5

6

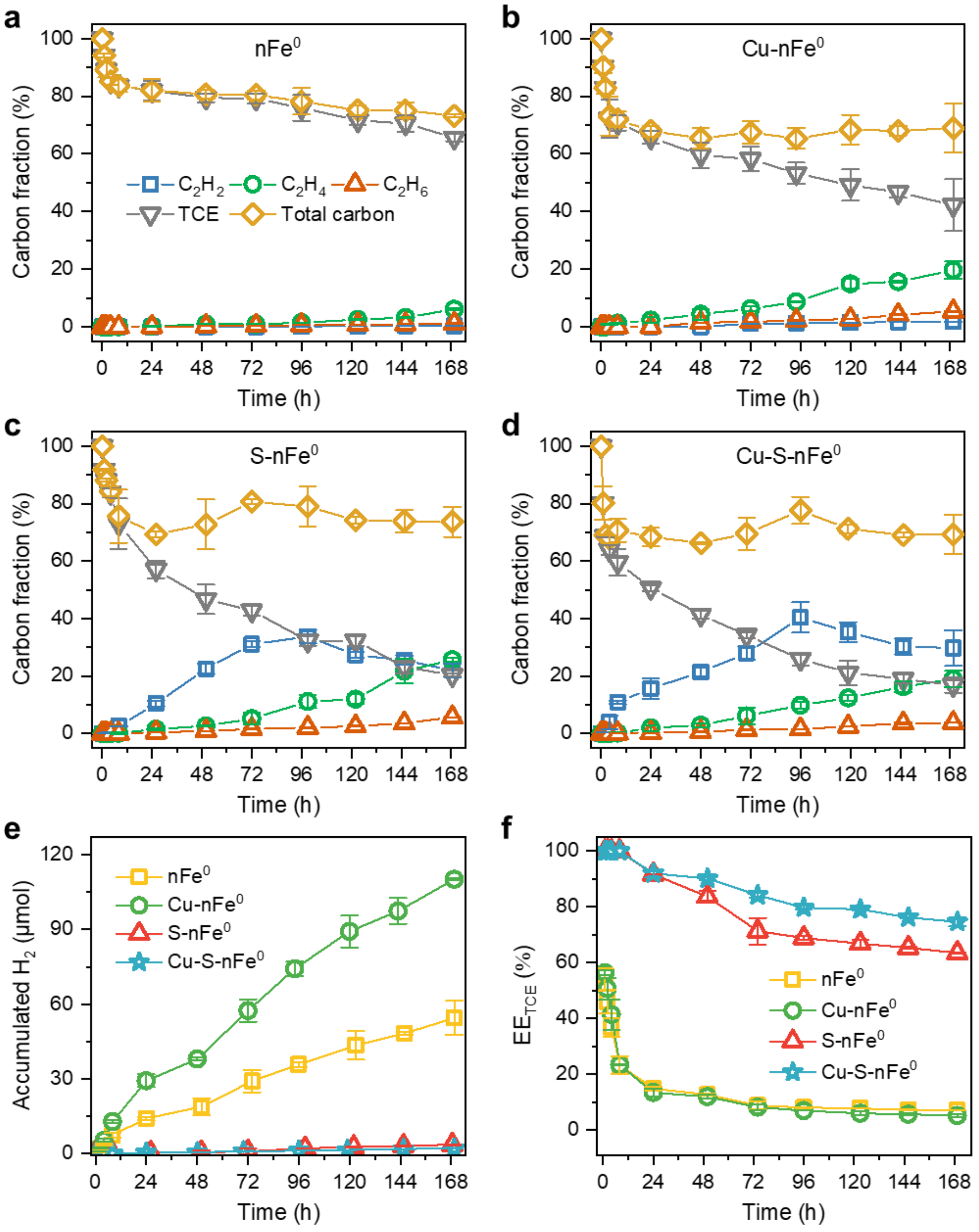

Figure S19. (a–d) Carbon fractions, (e) accumulated H2, and (f) electron efficiency (i.e., EE) during TCE removal in real groundwater by lattice S and/or Cu doped nFe^0 materials ($[Cu/Fe]_{dosed}$=1%, and $[S/Fe]_{dosed}$=0.14). Basic conditions are 1 g L^{-1} lattice-doped nFe^0 materials, 70 μM TCE, pH ≈ 8.5, temperature ≈ 25 °C, and a speed of 50 rpm. Data are presented as mean ± s.d. (n=3).

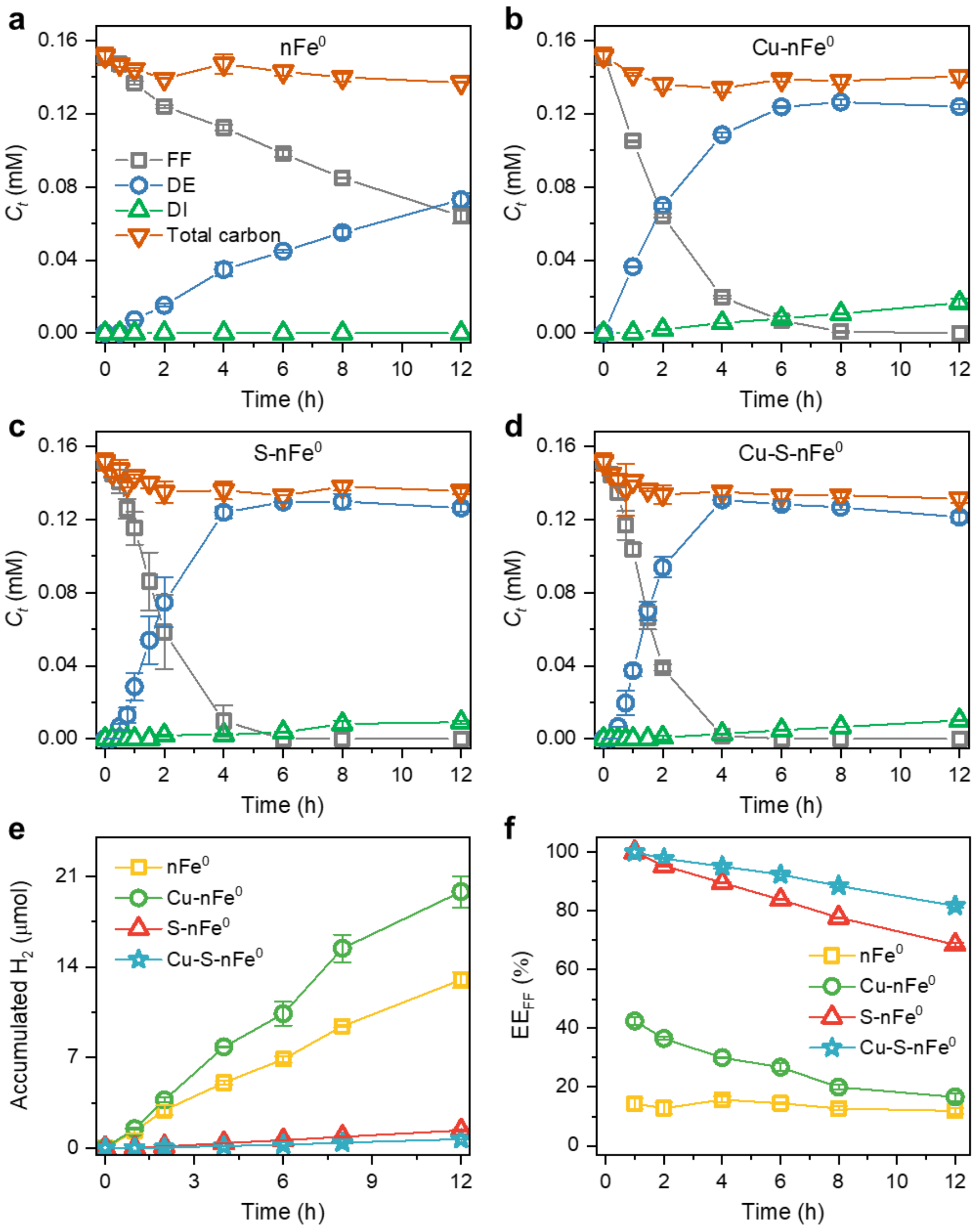

Figure S20. (a–d) Concentrations of FF and its degradation products, (e) accumulated H2, and (f) electron efficiency (i.e., EE) during FF removal in real groundwater by lattice S and/or Cu doped nFe^0 materials ($[Cu/Fe]_{dosed}$=1%, and $[S/Fe]_{dosed}$=0.14). Basic conditions are 1 g L^{-1} lattice-doped nFe^0 materials, 0.15 mM FF, pH ≈ 8.5, temperature ≈ 25 °C, and a speed of 50 rpm. Data are presented as mean ± s.d. (n=3). The abbreviation 'DE' and 'DI' is 'deschloro FF' and 'dideschloro FF', respectively.

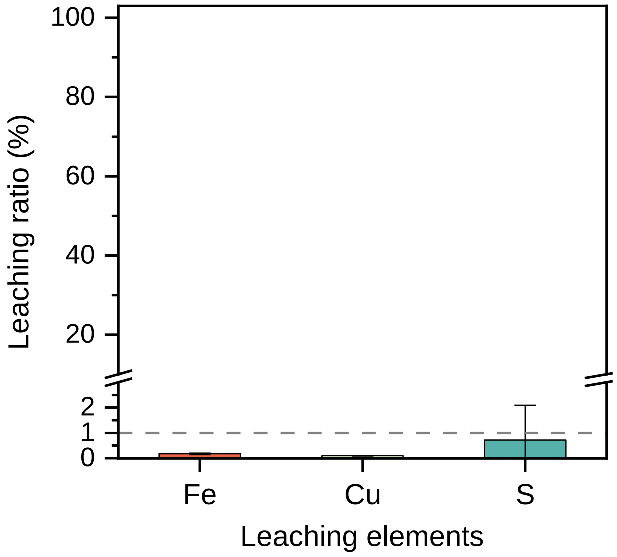

Figure S21. Leached ratio of Cu, Fe, and S elements in Cu-S-nFe^0 particles ($[Cu/Fe]_{dosed}$=1%, and $[S/Fe]_{dosed}$=0.14) after 20 days of TCE reaction in real groundwater. Basic conditions are 1 g L^{-1} lattice-doped nFe^0 materials, 70 μM TCE, pH ≈ 8.5, temperature ≈ 25 °C, and a speed of 50 rpm. Data are presented as mean ± s.d. (n=3).

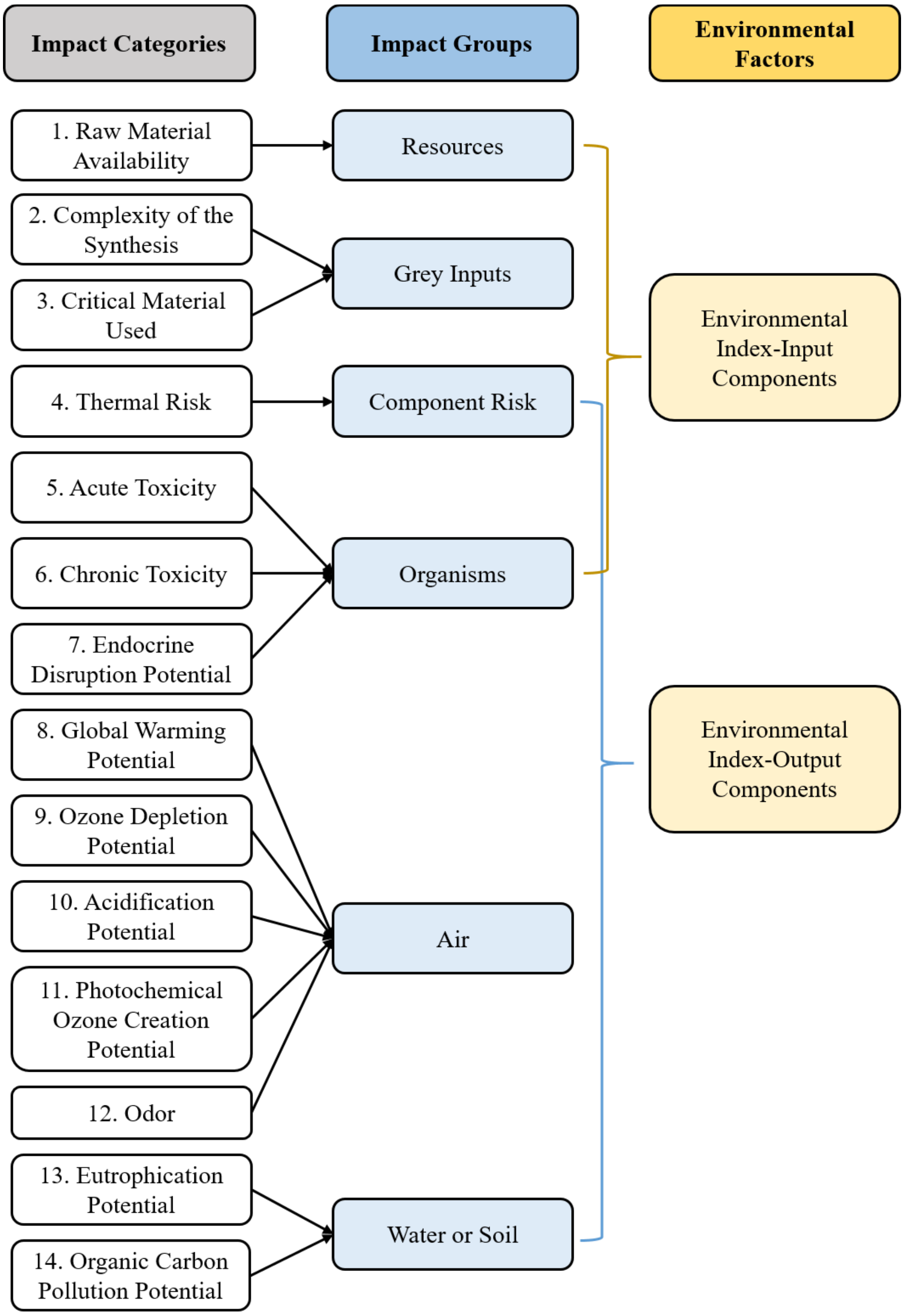

Scheme S1. Process flow for calculating environmental indices for inputs and outputs.[14]

4. Supplementary Tables

Table S1. Results of the shell fits of Fe K-edge EXAFS for a calculated $CuFeS_2$, FeS_2, $CuFe^0$ or Fe^0 along with the Cu-nFe^0 and Cu-S-nFe^0 samples.

Path		Coordination number							
Type	@-X	Calculated value				[Cu/Fe]			
		$CuFeS_2$	FeS_2	$CuFe^0$	Fe^0	Cu-nFe^0		Cu-S-nFe^0	
						0%	1%	0%	1%
Single scattering for cubic Fe^0	@Fe.1@	-	-	-	8	3.7	4.7	6.2	7.0
	@Fe.2@	-	-	-	6	2.0	2.7	4.0	5.5
	@Fe.3@	-	-	-	12	3.2	4.4	6.3	6.7
Single scattering for cubic Cu doped Fe^0	@Cu.1@	-	-	6	-	-	5.0	-	-
Single scattering for cubic pyrite FeS_2	@S.1@	-	6	-	-	-	-	1.5	2.1
	@Fe.4@	-	12	-	-	-	-	1.2	4.0
Single scattering for cubic chalcopyrite $CuFeS_2$	@Cu.2@	12	-	-	-	-	-	-	2.1
	@S.1@	12	-	-	-	-	-	-	6.1

Path		*R* (Å)							
Type	@-X	Calculated value				[Cu/Fe]			
		$CuFeS_2$	FeS_2	$CuFe^0$	Fe^0	Cu-nFe^0		Cu-S-nFe^0	
						0%	1%	0%	1%
Single scattering for cubic Fe^0	@Fe.1@	-	-	-	2.468	2.450	2.455	2.473	2.479
	@Fe.2@	-	-	-	2.850	2.790	2.789	2.837	2.834
	@Fe.3@	-	-	-	4.031	4.021	3.936	4.088	4.057
Single scattering for cubic Cu doped Fe^0	@Cu.1@	-	-	4.056	-	-	4.088	-	-
Single scattering for cubic pyrite FeS_2	@S.1@	-	3.453	-	-	-	-	3.679	3.780
	@Fe.4@	-	3.821	-	-	-	-	3.941	3.855
Single scattering for cubic chalcopyrite $CuFeS_2$	@Cu.2@	3.697	-	-	-	-	-	-	3.899
	@S.1@	4.335	-	-	-	-	-	-	4.328

Note: *R*, the distance between absorber and backscatter atoms.

R-factor (goodness of fit), σ^2 (Debye-Waller factor), and |ΔE| (inner potential correction) is below 0.02, 0.01 and 10, respectively.

Table S2. General Effect Index (GEI) calculated for synthesis of Cu-S-nFe^0.

Input												
Item	Mass index (kg kg−1)	Impact factors							Environmental factor (kg^{-1})		Environmental index (kg^{-1})	
		1	2	3	4	5	6	7	EF_{Mw}	EF_{Mult}	EI_{Mw}	EI_{Mult}
$FeCl_3$	2.90	C	C	C	C	B	C	B	0.075	1.300	0.217	3.770
$NaBH_4$	3.40	C	B	C	A	B	C	C	0.400	6.760	1.360	22.984
$CuCl_2{\cdot}2H_2O$	0.06	C	C	C	C	A	B	A	0.250	4.000	0.015	0.240
$Na_2S_2O_4$	0.22	C	B	B	B	B	C	C	0.225	2.197	0.049	0.483
Water	10.00	C	C	C	C	C	C	C	0.000	1.000	0.000	10.000
N_2	0.0001	C	C	C	C	C	C	C	0.000	1.000	0.000	0.0001
Total	16.580										1.642	37.477
GEI (kg)											0.099	2.260

Output														
Item	Mass index (kg kg^{-1})	Impact factors											Environmental factor (kg^{-1})	
		4	5	6	7	8	9	10	11	12	13	14	EF_{Mw}	EF_{Mult}
Cu-S-nFe^0	1.000	C	A	B	A	C	C	C	C	C	C	C	0.250	4.000
Losses	5.060	C	B	C	C	C	C	C	C	B	C	C	0.150	1.690
H_2S	0.070	A	A	C	A	C	C	C	C	B	C	C	0.575	20.800
H_2	0.450	A	C	C	C	C	C	C	C	C	C	C	0.250	4.000
N_2	0.0001	C	C	C	C	C	C	C	C	C	C	C	0.000	1.000
Water	10.000	C	C	C	C	C	C	C	C	C	C	C	0.000	1.000
Total	16.580													
GEI (kg)														

Output														
Item	Mass index (kg kg^{-1})	Impact factors											Environmental index (kg^{-1})	
		4	5	6	7	8	9	10	11	12	13	14	EI_{Mw}	EI_{Mult}
Cu-S-nFe^0	1.000	C	B	C	C	C	C	C	C	C	C	C	0.250	4.000
Losses	5.060	C	B	C	C	C	C	C	C	B	C	C	0.759	8.551
H_2S	0.070	A	A	C	A	C	C	C	C	B	C	C	0.040	1.456
H_2	0.450	A	C	C	C	C	C	C	C	C	C	C	0.113	1.800
N_2	0.0001	C	C	C	C	C	C	C	C	C	C	C	0.000	0.0001
Water	10.000	C	C	C	C	C	C	C	C	C	C	C	0.000	10.000
Total	16.580												1.162	25.808
GEI (kg)													0.070	1.557

Note: Items 1-14 correspond to 14 environmental impact categories (see Scheme S1). A, B, and C represent the degree of influence of each condition on different environmental categories, with the relationship of influence being A > B > C. The EF_{Mult} uses the values A = 4, B = 1.3, and C = 1, and these values are aggregated by multiplication. Consequently, possible values of EF_{Mult} range from 1 to 256. The alternative EF_{Mw} employs the values A = 1, B = 0.3, and C = 0. There, the EF_{Mw} is calculated by averaging. Its values range between 0 and 1. The lower the GEI, the less environmental impact it has.[14]

Table S3. Material usage cost of different materials for 1 m^3 remedation.

Material	Synthesis cost ($/g)	Electronic selectivity	Removal ratio	Material usage (g)	Total cost ($)
**TCE**					
nFe^0	2.73	0.07	0.34	164.71	449.57
$Cu\text{-}nFe^0$	2.74	0.05	0.58	135.17	370.91
$S\text{-}nFe^0$	2.74	0.64	0.80	7.66	20.95
$Cu\text{-}S\text{-}nFe^0$	2.75	0.75	0.83	6.30	17.32

Material	Synthesis cost ($/g)	Electronic selectivity	Removal ratio	Material usage (g)	Total cost ($)
**FF**					
nFe^0	2.73	0.12	0.58	122.09	333.26
$Cu\text{-}nFe^0$	2.74	0.20	0.99	42.70	117.18
$S\text{-}nFe^0$	2.74	0.89	0.99	9.64	26.39
$Cu\text{-}S\text{-}nFe^0$	2.75	0.95	0.94	9.52	26.18

Table S4. Properties of the groundwater sample.

Parameters	Values	Unit
pH	8.4 ± 0.2	/
UV_{254}	0.053	/
TOC	1.72 ± 0.03	mg C/L
Cl^{-}	2.32 ± 0.04	mg/L
SO_4^{2-}	28.50 ± 0.50	mg/L
NO_3^{-}	5.02 ± 0.08	mg/L
PO_4^{3-}	5.40 ± 0.03	mg/L
Na^{+}	29.01 ± 0.80	mg/L
K^{+}	20.37± 1.15	mg/L
Mg^{2+}	6.89 ± 0.81	mg/L
Ca^{2+}	39.05 ± 3.27	mg/L

Table S5. Input chemical and energy costs of 1 kg Cu-S-nFe^0 production.

Chemcial & energy	Price	Amount	Total cost ($)
$FeCl_3$	23.53 $/kg	2.90 kg	68.24
$NaBH_4$	702.69 $/kg	3.40 kg	2389.15
$CuCl_2 \cdot 2H_2O$	238.02 $/kg	0.06 kg	14.28
$Na_2S_2O_4$	30.99 $/kg	0.22 kg	6.82
Water	0.65 $/m^3	0.01 m^3 (wash)	0.065
N_2	0.21 $/L	0.10 L	0.02
Electricity power	0.07 $/kWh	3665 kWh (stir & dry)	271.59

Note: this is based on lab synthesis.

5. References

[1] a) E. J. Kim, J. H. Kim, A. M. Azad, Y. S. Chang, *ACS Appl. Mater. Inter.* **2011**, *3*, 1457−1462; b) X. H. Hu, C. H. Chen, D. Chen, V. Noël, H. Oji, S. Ghoshal, G. V. Lowry, P. G. Tratnyek, D. H. Lin, L. Z. Zhu, J. Xu, *Nat. Water* **2024**, *2*, 84−92.

[2] B. Ravel, M. Newville, *J. Synchrotron. Radiat.* **2005**, *12*, 537−541.

[3] J. Xu, A. Avellan, H. Li, X. T. Liu, V. Noel, Z. M. Lou, Y. Wang, R. Kaegi, G. Henkelman, G. V. Lowry, *Adv. Mater.* **2020**, *32*, 1906910.

[4] A. R. Lennie, S. A. T. Redfern, P. F. Schofield, D. J. Vaughan, *Mineral. Mag.* **1995**, *59*, 677−683.

[5] E. P. Abrahamson, S. L. Lopata, *T. Metall. Soc. Aime.* **1966**, *76*, 4579002.

[6] C. L. Burdick, J. H. Ellis, *J. Am. Chem. Soc.* **1917**, *39*, 2518−2525.

[7] a) J. Xu, Y. Wang, C. Weng, W. L. Bai, Y. Jiao, R. Kaegi, G. V. Lowry, Environ. Sci. Technol. **2019**, 53, 5936−5945; b) J. Xu, Z. Cao, H. Zhou, Z. M. Lou, Y. Wang, X. H. Xu, G. V. Lowry, Environ. Sci. Technol. **2019**, 53, 13344−13352.

[8] a) L. B. H. B. Hammer, J. K. Nørskov, *Phys. Rev. B* **1999**, *59*, 7413−7421; b) G. Kresse, J. Furthmuller, *Phys. Rev. B* **1996**, *54*, 11169−11186; c) G. Kresse, J. Furthmuller, *Comp. Mater. Sci.* **1996**, *6*, 15−50.

[9] P. E. Blochl, *Phys. Rev. B* **1994**, *50*, 17953−17979.

[10] S. Grimme, *J. Comput. Chem.* **2006**, *27*, 1787−1799.

[11] H. J. Monkhorst, J. D. Pack, *Phys. Rev. B* **1976**, *13*, 5188−5192.

[12] a) Z. Cao, X. Liu, J. Xu, J. Zhang, Y. Yang, J. L. Zhou, X. H. Xu, G. V. Lowry, *Environ. Sci. Technol.* **2017**, *51*, 11269−11277; b) S. C. Cai, B. Chen, X. J. Qiu, J. M. Li, P. G. Tratnyek, F. He, *Environ. Sci. Technol.* **2021**, *55*, 645−654; c) F. X. Meng, J. Xu, H. W. Dai, Y. L. Yu, D. H. Lin, *Environ. Sci. Technol.* **2022**, *56*, 4489−4497.

[13] C. H. Chen, Q. H. Zhou, Z. Y. Guo, H. Li, C. Miao, D. Chen, X. H. Hu, X. Feng, V. Noël, S. Ghoshal, G. V. Lowry, L. Z. Zhu, D. H. Lin, J. Xu, *Nat. Sustain.* **2024**, *7*, 1264–1272.

[14] A. Biwer, E. Heinzle, *J. Chem. Technol. Biot.* **2004**, *79*, 597−609.

[15] X. Y. Wang, Y. J. Chen, F. Li, R. K. Miao, J. E. Huang, Z. L. Zhao, X. Y. Li, R. Dorakhan, S. L. Chu, J. H. Wu, S. X. Zheng, W. Y. Ni, D. Kim, S. Park, Y. X. Liang, A. Ozden, P. F. Ou, Y. Hou, D. Sinton, E. H. Sargent, *Nat. Commun.* **2024**, *15*, 616.

6. Author Contributions

Xiaohong Hu and Jiang Xu proposed the research idea.
Jiang Xu, Daohui Lin, Jie Hou, and Lizhong Zhu supervised the project.
Xiaohong Hu conducted the experiments and analyzed the data.
Qianhai Zhou and Chaohuang Chen assisted with environmental friendliness and techno-economic assessments.
Du Chen and Yiman Gao contributed to material synthesis.
Vincent Noël supported the crystalline structure analysis.
Zhongyuan Guo performed the DFT calculations and modeling.
Xiaohong Hu and Jiang Xu analyzed the results and drafted the manuscript.
All authors discussed the results and approved the final version of the manuscript.
